# The Effects of Prenatal Dexamethasone Exposure on Brain Metabolic Homeostasis in Adulthood: Implications for Depression

**DOI:** 10.3390/ijms24021156

**Published:** 2023-01-06

**Authors:** Katarzyna Głombik, Magdalena Kukla-Bartoszek, Katarzyna Curzytek, Jan Detka, Agnieszka Basta-Kaim, Bogusława Budziszewska

**Affiliations:** Laboratory of Immunoendocrinology, Department of Experimental Neuroendocrinology, Maj Institute of Pharmacology, Polish Academy of Sciences, Smętna 12, 31-343 Kraków, Poland

**Keywords:** brain, depression, animal model, dexamethasone, bioenergetics

## Abstract

Since depression produces a long-term negative impact on quality of life, understanding the pathophysiological changes implicated in this disorder is urgent. There is growing evidence that demonstrates a key role for dysfunctional energy metabolism in driving the onset of depression; thus, bioenergetic alterations should be extensively studied. Brain metabolism is known to be a glucocorticoid-sensitive process, but the long-lasting consequences in adulthood following high levels of glucocorticoids at the early stages of life are unclear. We examined a possible association between brain energetic changes induced by synthetic glucocorticoid-dexamethasone treatment in the prenatal period and depressive-like behavior. The results show a reduction in the oxidative phosphorylation process, Krebs cycle impairment, and a weakening of the connection between the Krebs cycle and glycolysis in the frontal cortex of animals receiving dexamethasone, which leads to ATP reduction. These changes appear to be mainly due to decreased expression of pyruvate dehydrogenase, impairment of lactate transport to neurons, and pyruvate to the mitochondria. Acute stress in adulthood only slightly modified the observed alterations in the frontal cortex, while in the case of the hippocampus, prenatal exposure to dexamethasone made this structure more sensitive to future adverse factors.

## 1. Introduction

Mental disorders refer to a wide range of mental health conditions that are among the most important health problems of the 21st century, and among them, the soaring incidence of depression is a serious challenge to contemporary medicine [1]. Despite many years of research and a variety of pharmacological approaches [2], this disease still produces a long-term negative impact on the quality of life and a significant reduction in life expectancy. Depression is a complex disorder characterized by alterations in several biological systems, including genetic, neuroendocrine, biological, psychosocial, and environmental interactions. Among the factors that raise the incidence of depression, stress plays a particularly important role. Stress exerts many effects on the central nervous system (CNS) and can cause structural changes in different parts of the brain [3] that have long-term effects, which have been shown in both preclinical and clinical studies [4]. By activating the hypothalamic–pituitary–adrenal (HPA) axis, stress affects neurotransmission, the immune system, and metabolism and leads to disturbances in the function of CNS cells. The observed neurotransmission deficits in the course of depression are accompanied by an array of other changes that together cause the manifestation of depressive symptoms. It is well known that the prenatal period is especially sensitive to traumatic stress stimuli, and during this period, environmental stress may have adverse effects on offspring and increase the risk of depression during adulthood. However, the nature of this link has not been comprehensively studied, and several mechanisms must be taken into account. Many lines of evidence from human studies indicate that exposure to excessive levels of stress hormones, glucocorticoids (GCs), significantly increases the risk of depression later in life [5].

The GCs are steroid hormones that act via mineralocorticoid receptors (MRs) and glucocorticoid receptors (GRs) [6]. The MR has a high affinity for GCs and plays a pivotal role during basal physiological conditions, whereas the GR, a transcriptional regulator, is characterized by a reduced affinity for GCs and responsiveness when their levels are high, which is common in depression [7]. Disturbances in GR function, mostly in the prefrontal cortex, hippocampus, and hypothalamus, are considered to be involved in the development of depression among those with impaired GR expression/function and HPA axis hyperactivity, both in humans and animal models [8,9]. GCs play an important role in neuronal survival processes, neurogenesis, cognitive function, memory formation, and inflammatory processes [10], and severe or chronic stress exposure at different periods of life can disrupt these processes, cause neural cell death and atrophy of neuronal processes, and affect neurogenesis and plasticity. Moreover, the effects of GCs on brain structure and function vary across the lifespan.

It has been shown that exposure of the brain to high levels of GCs at early stages of life may have long-lasting and dramatic consequences on later HPA function and other glucocorticoid-sensitive processes, such as metabolism, in humans and rodents [11]. Adverse events in early life (which often result in GC elevation) may exert similar effects [12]. For example, the widely used, potent GR agonist synthetic glucocorticoid dexamethasone (DEX) treatment in the prenatal period leads to long-term changes in HPA axis activity and in the level of neurotransmitters [13]. However, the World Health Organization guidelines for procedures followed when there is a risk of a premature delivery recommend antenatal corticosteroid therapy in cases of threatened premature birth between 24 and 34 weeks of pregnancy. In such cases, synthetic glucocorticoids (sGCs) are administered to accelerate the maturation of the alveolar epithelium and to stimulate respiratory system maturation in the fetus [14]. Notwithstanding, there is evidence from animal models that antenatal corticosteroid treatment may have an adverse impact on the development of the immature brain by inhibiting growth factors and facilitating apoptosis [15]. However, the effects of exposure to this steroid in the prenatal period on the induction of permanent metabolic alterations in the brain and the mechanism responsible for the majority of changes observed in the offspring remain unclear.

The long-lasting effects of GCs are particularly worth investigating because currently, great importance is attached to metabolic disturbances in CNS cells, which may be one of the main causes of depression [16]. Glucose metabolism provides fuel for physiological brain function through the generation of ATP. Energy is necessary for the proper function of neurons including the bioenergetic basis for neurotransmission, scavenging of free radicals, regulation of cell death and calcium homeostasis [17,18]. Inadequate energy supply and bioenergetic deficits affect almost every level of the cellular or biochemical cascades and predispose individuals to a variety of brain disorders, such as depression.

**Figure 1:**
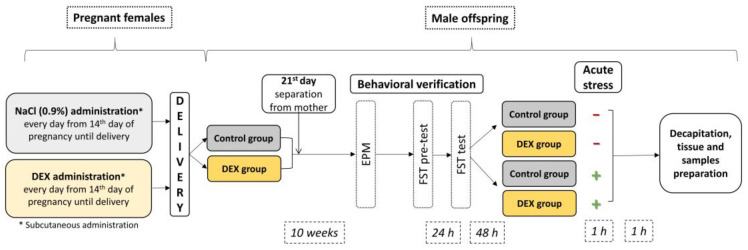
Schematic diagram of the experiment.

Thus, we investigated whether third-trimester prenatal DEX treatment is associated with metabolic alterations in the brain at adult age using a rat model of depression (Figure 1). In this study, we used rats prenatally exposed to DEX as a model of depression, which is well characterized. Existing studies on humans and rodents have shown that repeated exposure to DEX in late gestation leads to depression-like behavior, increased HPA axis activity, and immune system alterations in adult offspring, and this phenotype can be reversed by antidepressant drug treatment [19,20,21,22]. Our aim was to determine not only the impact of DEX on behavioral changes but also on the glycolysis, Krebs cycle, oxidative phosphorylation, and insulin signaling pathway in the brain to bring us closer to understanding the background of depression-like deficits. In the first phase of the experiment, we asked ourselves whether there is an impairment of ATP production in the process of oxidative phosphorylation in animals treated with DEX in the prenatal period, in two brain structures involved in the pathomechanisms of depression: in the frontal cortex and the hippocampus. Then we tried to trace the energy production in the brain also at other stages and found the mechanisms behind the DEX-induced changes. At this stage, we additionally included groups of animals stressed in adulthood, as the structures and processes we study may be sensitive to this factor.

## 2. Results

### 2.1. Behavioral Studies

#### The Effects of Prenatal Dexamethasone Treatment on the Behavior of Rats

**Figure 2:**
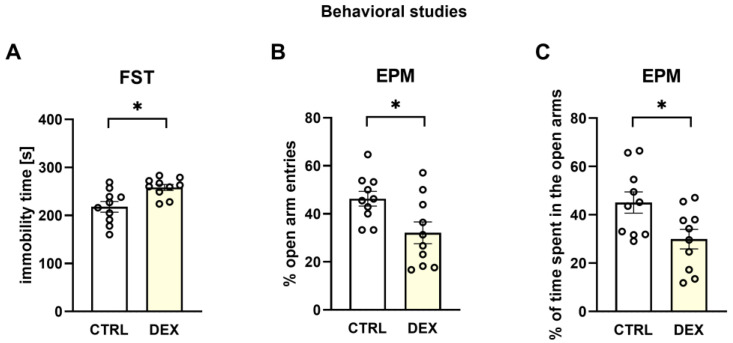
The effects of prenatal dexamethasone treatment on the immobility time (**A**) measured in the FST, % of open arm entries (**B**) and % of time spent in the open arms (**C**) of the EPM; * *p* < 0.05, *n* = 10. The results are expressed as the mean ± SEM. Statistics: one-way ANOVA.

To assess depression-like and anxiety-like behavior, we performed a forced swim test (FST) and an elevated plus maze (EPM) test, respectively. In the FST, animals whose mothers were treated with DEX during pregnancy were characterized by significant changes in the immobility time—the measured time was longer compared to the Control group (DEX effect F_1,18_ = 10.19; *p* = 0.005) (Figure 2A). In the EPM test, prenatal treatment with DEX caused a significant decrease in the percentage of open arm entries (DEX effect F_1,18_ = 6.74; *p* = 0.018) (Figure 2B) and a reduction in the percentage of time spent in the open arms (DEX effect F_1,18_ = 6.39; *p* = 0.021) (Figure 2C) by the rats.

### 2.2. Biochemical Studies

#### 2.2.1. The Effects of Dexamethasone Treatment on Oxidative Phosphorylation Complex (OXPHOS) Levels in the Mitochondria-Enriched Fraction of the Frontal Cortex and Hippocampus

**Figure 3:**
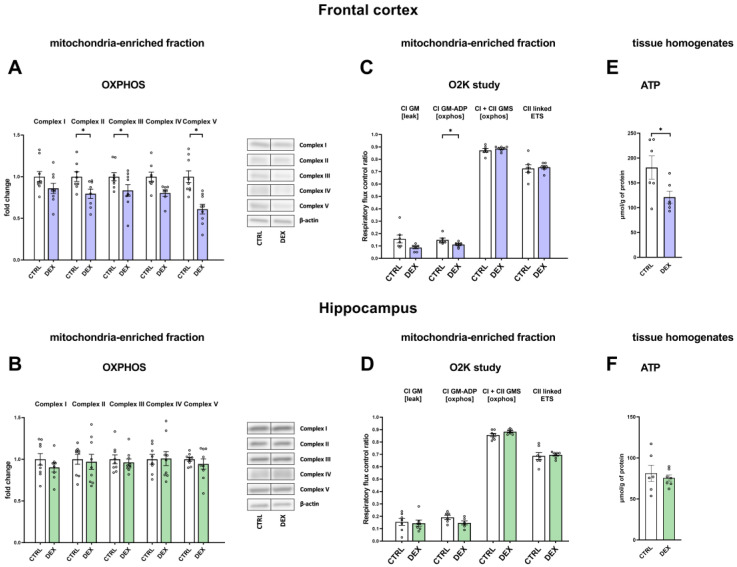
The effects of DEX treatment on OXPHOS levels, mitochondrial respiration capacity, and ATP levels in the mitochondria-enriched fraction/homogenates of the frontal cortex (**A**,**C**,**E**) and the hippocampus (**B**,**D**,**F**); OXPHOS levels were determined with Western blotting analysis, mitochondrial respiration capacity was determined via respirometry, and ATP level via luminescent measurements; * *p* < 0.05, *n* = 7–9 for OXPHOS determination, *n* = 6–7 for O2K study and for ATP level assessment. The results are expressed as the average fold change ± SEM (**A**,**B**) or as the mean ± SEM (**C**–**F**). Statistics: one-way ANOVA.

To evaluate the effects of prenatal administration of DEX on OXPHOS complex levels, we carried out Western blotting analysis of the mitochondria-enriched fractions isolated from the frontal cortices and hippocampi of the investigated animals. Analysis showed decreased levels of complex II (DEX effect F_1,14_ = 6.49; *p* = 0.023), complex IV (DEX effect F_1,13_ = 7.99; *p* = 0.014), and complex V (DEX effect F_1,16_ = 18.07; *p* = 0.001) in the frontal cortices of rats prenatally treated with DEX (Figure 3A). There were no changes in the expression of mitochondrial complexes in the hippocampi of these animals (Figure 3B).

#### 2.2.2. The Effects of Dexamethasone Treatment on Mitochondrial Respiration Capacity in the Mitochondria-Enriched Fraction of the Frontal Cortex and Hippocampus

To determine the effect of DEX treatment on mitochondrial respiration capacity, we performed respirometry measurements. In the frontal cortex, the ADP-stimulated (CI GM-ADP) state was decreased in prenatal DEX-treated animals (DEX effect F_1,10_ = 5.44; *p* = 0.042) (Figure 3C), whereas no changes were detected in measured states in the hippocampus (Figure 3D).

#### 2.2.3. The Effects of Dexamethasone Treatment on ATP Production in the Frontal Cortex and Hippocampal Tissue Homogenates

Due to the changes in the levels of proteins in the respiratory complexes and mitochondrial respiration capacity, we decided to study whether the observed deficits also caused disturbances in the production of ATP in the brain.

As shown in Figure 3E,F, DEX administered during late gestation to the mothers resulted in the diminished production of ATP in the frontal cortices of the offspring in adulthood (DEX effect F_1,10_ = 5.18; *p* = 0.046), whereas the treatment used did not affect the level of measured biochemical energy storage in the hippocampi.

Since we observed dysregulation in the oxidative phosphorylation process and ATP synthesis, in the next stages of the study, we decided to investigate whether there were changes in the key enzymes of glycolysis, in the enzyme that connects glycolysis with the Krebs cycle (pyruvate dehydrogenase) and what mechanism may be responsible for the observed changes in the depression model used. Moreover, we decided to check whether the administration of DEX prenatally evoked metabolic changes not only under basal conditions but also altered brain sensitivity to stress in adult life; therefore, some of the Control and DEX animals were subjected to hourly immobilization stress one hour before tissue collection.

#### 2.2.4. The Effects of Dexamethasone Treatment and Acute Stress on the Levels of Glycolytic Enzymes (Hexokinase, Phosphofructokinase, and Pyruvate Kinase) in the Frontal Cortex and Hippocampal Tissue Homogenates

**Figure 4:**
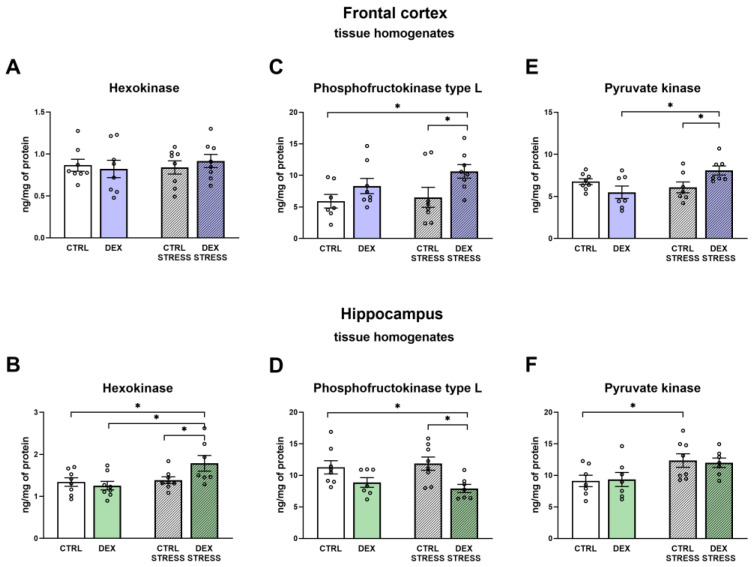
The effects of DEX treatment and acute stress on the levels of enzymes hexokinase, phosphofructokinase, and pyruvate kinase in tissue homogenates of the frontal cortex (**A**,**C**,**E**) and the hippocampus (**B**,**D**,**F**); enzyme levels were determined with ELISA assays; * *p* < 0.05, *n* = 7–8. The results are expressed as the mean ± SEM. Statistics: two-way ANOVA, followed by the Duncan test.

Hexokinase level in the frontal cortex did not differ between the investigated groups (Figure 4A), but in the hippocampus of rats prenatally treated with DEX and subjected to stress in adulthood (Stress effect F_1,27_ = 5.80; *p* = 0.023), a greater level of this enzyme vs. other groups was observed (Figure 4B).

When the protein level of phosphofructokinase was measured, the effect of DEX was observed in both brain structures (DEX effect F_1,27_ = 6.60; *p* = 0.016 for frontal cortex, and DEX effect F_1,26_ = 11.88; *p* = 0.002 for hippocampus) (Figure 4C,D). In the frontal cortex, the level of the enzyme was increased in the DEX Stress group of animals when compared to both Control groups (Figure 4C). The opposite effect was demonstrated in the hippocampus, where the level of phosphofructokinase in the DEX Stress group was significantly decreased compared to both Control groups (Figure 4D).

The pyruvate kinase level was shown to be higher in the frontal cortex in the DEX Stress group than in the DEX and Control groups subjected to acute stress (DEX × Stress effect F_1,25_ = 9.12; *p* = 0.006; Stress effect F_1,25_ = 4.38; *p* = 0.047) (Figure 4E). In the hippocampus, stress itself induced the growth of pyruvate kinase in Control rats (Stress effect F_1,26_ = 7.12; *p* = 0.013) (Figure 4F).

#### 2.2.5. The Effects of Dexamethasone Treatment and Acute Stress on the Levels of Pyruvate and Lactate in the Cytosolic and Mitochondria-Enriched Fraction of the Frontal Cortex and Hippocampus

**Figure 5:**
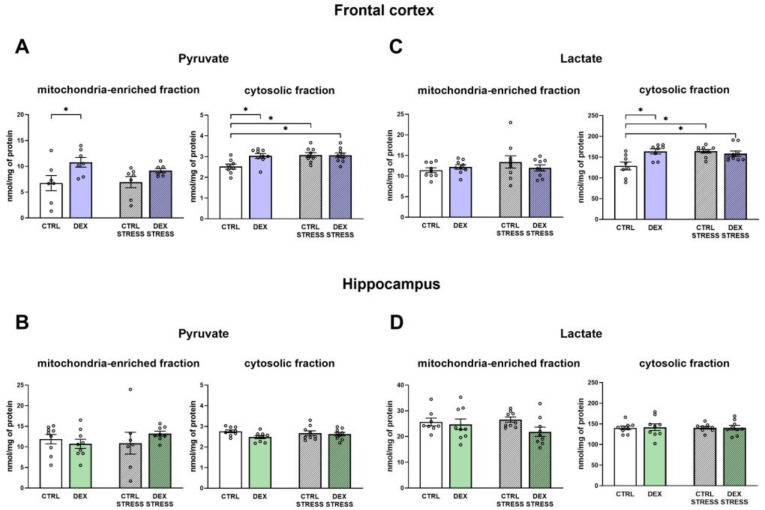
The effects of DEX treatment and acute stress on pyruvate and lactate levels in the mitochondria-enriched and cytosolic fractions of the frontal cortex (**A**,**C**) and the hippocampus (**B**,**D**); lactate and pyruvate levels were determined via colorimetric and fluorometric measurements, respectively; * *p* < 0.05, *n* = 7–9. The results are expressed as the mean ± SEM. Statistics: two-way ANOVA, followed by the Duncan test.

In the frontal cortex, significant changes in the level of pyruvate were observed in both cellular fractions. In the DEX group, pyruvate was increased in the mitochondria (DEX effect F_1,24_ = 8.94; *p* = 0.006) and cytosol (DEX effect F_1,31_ = 4.55; *p* = 0.041) (Figure 5A). Additionally, in Control and DEX-treated animals subjected to acute stress as adults, the pyruvate concentration in the cytosol was higher than that in the Control (Stress effect F_1,31_ = 6.27; *p* = 0.018; DEX × Stress effect F_1,31_ = 5.22; *p* = 0.029) (Figure 5A).

In the frontal cortex, lactate level was increased by DEX and/or stress in all examined groups (DEX effect F_1,30_ = 4.74; *p* = 0.038; Stress effect F_1,30_ = 4.95; *p* = 0.034; DEX × Stress effect F_1,30_ = 9.37; *p* = 0.005) in the cytosolic fraction (Figure 5C). No change in lactate level was observed in the mitochondrial fraction in this brain structure.

In the hippocampus, pyruvate and lactate levels did not differ between the investigated groups, either in the case of mitochondria or in the cytosolic fraction (Figure 5B,D).

#### 2.2.6. The Effects of Dexamethasone Treatment and Acute Stress on Pyruvate Dehydrogenase (PDH) Levels and Activity in the Mitochondria-Enriched Fraction of the Frontal Cortex and Hippocampus

**Figure 6:**
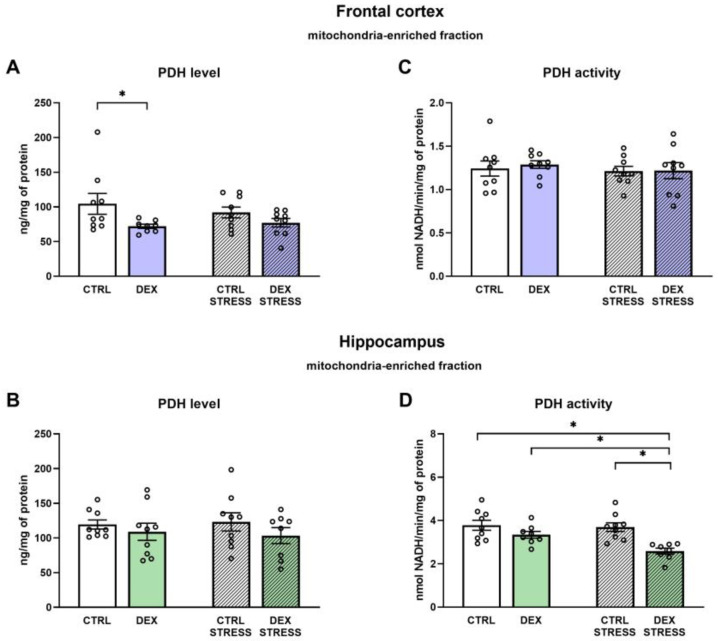
The effects of DEX treatment and acute stress on pyruvate dehydrogenase (PDH) level and activity in the mitochondria-enriched fraction of the frontal cortex (**A**,**C**) and the hippocampus (**B**,**D**); PDH level was determined with ELISA assay, while activity with colorimetric assay; * *p* < 0.05, *n* = 8–9. The results are expressed as the mean ± SEM. Statistics: two-way ANOVA, followed by the Duncan test.

The level of PDH was decreased in the frontal cortex of DEX-treated animals (DEX effect F_1,31_ = 6.37; *p* = 0.017) vs. the Control group, but there were no changes in the activity of this enzyme (Figure 6A,C). In the hippocampus, despite the lack of differences in the level of PDH between the groups, the measured enzyme activity was decreased in the DEX Stress group vs. Control, vs. DEX and vs. Control Stress groups (DEX effect F_1,30_ = 16.74; *p* = 0.001 and Stress effect F_1,30_ = 4.92, *p* = 0.003) (Figure 6B,D).

#### 2.2.7. The Effects of Dexamethasone Treatment and Acute Stress on Hexokinase-1 (HK1) and Voltage-Dependent Anion-Selective Channel 1 (VDAC1) Protein Levels in the Mitochondria-Enriched Fraction of the Frontal Cortex and Hippocampus

One of the possible causes of observed deficits in brain energy metabolism is the dysregulation in the process of detachment of mitochondrial hexokinase from the outer mitochondrial membrane, which affects the activity of this enzyme. An essential protein in this process is VDAC1, a channel that binds hexokinase to the mitochondrial membrane. Therefore, in this step, we investigated the impact of DEX and stress on HK1 and VDAC1 levels in the mitochondria-enriched fraction.

**Figure 7:**
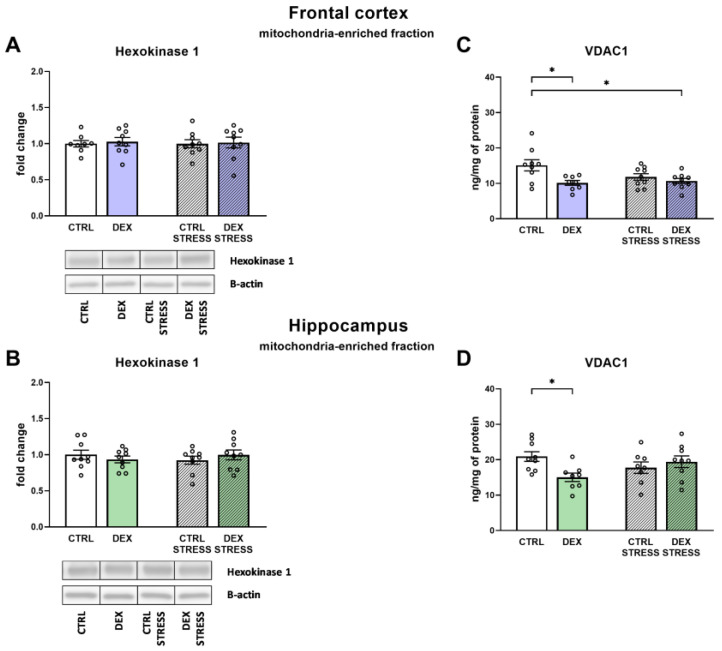
The effects of DEX treatment and acute stress on the levels of hexokinase-1 (HK1) and voltage-dependent anion-selective channel 1 (VDAC1) in the mitochondria-enriched fraction of the frontal cortex (**A**,**C**) and the hippocampus (**B**,**D**); HK1 level was determined with Western blotting analysis, and VDAC1 with ELISA assay; * *p* < 0.05, *n* = 8–9. The results are expressed as the average fold change ± SEM (**A**,**B**) or as the mean ± SEM (**C**,**D**). Statistics: two-way ANOVA, followed by the Duncan test.

No changes in HK1 were detected in either examined brain area (Figure 7A,B). In the frontal cortex, the level of VDAC1 was demonstrated to be decreased in the prenatally DEX-treated rats with/without stress application when compared to Control animals (DEX effect F_1,31_ = 8.30; *p* = 0.007) (Figure 7C). Studies in the hippocampus indicated that the level of the investigated channel was lower only in the DEX group without additional stress (DEX × Stress effect F_1,30_ = 6.54; *p* = 0.016) (Figure 7D).

#### 2.2.8. The Effects of Dexamethasone Treatment and Acute Stress on Monocarboxylate Transporters (MCT2 and MCT4) and Mitochondrial Pyruvate Carriers (MPC1 and MPC2) in the Frontal Cortex and Hippocampal Tissue Homogenates

Because the disruption in ATP production may result from the reduced transport of lactate from astrocytes to the neurons and/or pyruvate into the mitochondria, we studied the possible involvement of changes of their monocarboxylate transporters and carriers in these processes by assessing protein levels.

**Figure 8:**
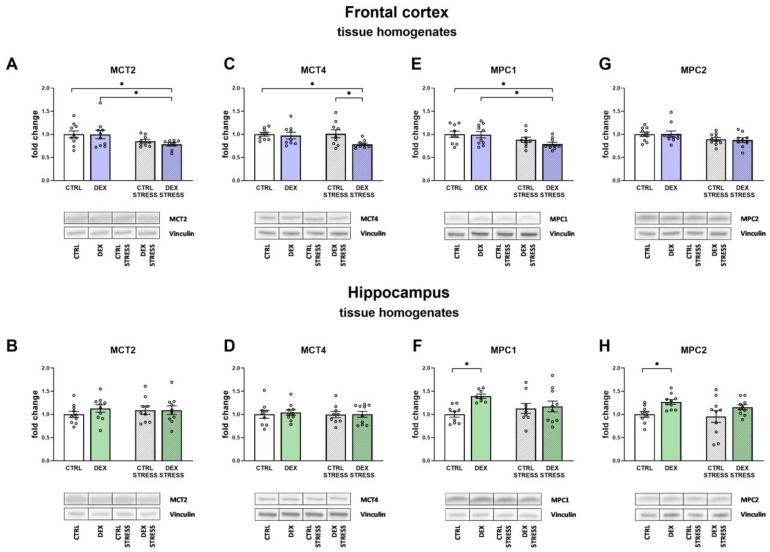
The effects of DEX treatment and acute stress on the levels of MCT2, MCT4, MPC1, and MPC2 in the tissue homogenates of the frontal cortex (**A**,**C**,**E**,**G**) and the hippocampus (**B**,**D**,**F**,**H**); all proteins levels were determined with Western blotting analysis; * *p* < 0.05, *n* = 8–10. The results are expressed as the average fold change ± SEM. Statistics: two-way ANOVA, followed by the Duncan test.

Significant changes in the level of MCT2 and MCT4 were demonstrated in the frontal cortex. Stress applied to animals whose mothers were treated with DEX during pregnancy caused a reduction in MCT2 protein level (Stress effect F_1,33_ = 6.95; *p* = 0.013) when compared to Control and DEX-treated animals (Figure 8A). Similarly, MCT4 level was decreased in the same group of animals when compared to both Control groups (DEX effect F_1,32_ = 4.34; *p* = 0.045) (Figure 8C). The transporter levels were changed neither by DEX nor by stress in the hippocampus (Figure 8B,D).

Mitochondrial pyruvate carrier 1 (MPC1) was downregulated by stress only in the frontal cortex of prenatally DEX-treated animals (Stress effect F_1,33_ = 7.16; *p* = 0.011) (Figure 8E), whereas in the hippocampus of DEX-treated animals, the MPC1 level was increased when compared to the Control group (DEX effect F_1,32_ = 5.49; *p* = 0.025) (Figure 8F).

No changes in MPC2 protein level were detected in the frontal cortex (Figure 8G), but in the hippocampus, upregulation of this carrier was observed in the DEX-treated group vs. the Control group (DEX effect F_1,35_ = 8.16; *p* = 0.007) (Figure 8H).

#### 2.2.9. The Effects of Dexamethasone Treatment and Acute Stress on Lactate Receptor G-Protein-Coupled Receptor 81/Hydroxycarboxylic Acid Receptor 1 (GPR81) in the Frontal Cortex and Hippocampal Tissue Homogenates

Since it is known that lactate acts in the brain not only as a substrate but also as a signaling molecule by activating the GPR81 receptor, we decided to check if prenatal DEX administration modulates the level of this receptor.

**Figure 9:**
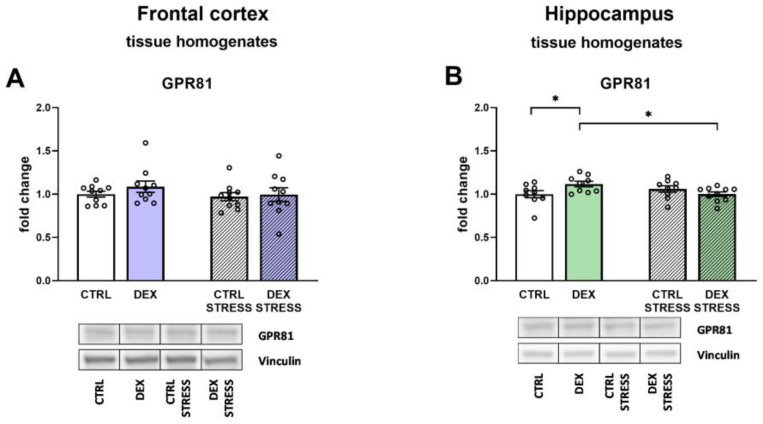
The effects of DEX treatment and acute stress on the level of GPR81 in tissue homogenates of the frontal cortex and (**A**) the hippocampus (**B**); protein level was determined with Western blotting analysis; * *p* < 0.05, *n* = 9–10. The results are expressed as the average fold change ± SEM. Statistics: two-way ANOVA, followed by the Duncan test.

No changes between the studied groups were detected, taking into account the level of GPR81 in the frontal cortex (Figure 9A), whereas in the hippocampus, prenatal administration of DEX resulted in an increase in receptor level; however, after acute stress in adulthood, the observed effect was reversed (DEX × Stress effect F_1,33_ = 6.70; *p* = 0.014) (Figure 9B).

#### 2.2.10. The Effects of Dexamethasone Treatment and Acute Stress on the Insulin Signaling Pathway in the Frontal Cortex and Hippocampal Tissue Homogenates

Changes in brain energy metabolism can also occur with disruption between mitochondrial function and insulin signaling pathway interplay. Because insulin action regulates brain mitochondrial function on almost all levels, including respiratory processes, protein homeostasis, and ATP production, we evaluated possible DEX-induced changes in the level of insulin receptor and/or its signal transduction network by studying the protein levels of insulin, insulin receptor (IR) total and phosphorylated form, insulin receptor substrate 1 (IRS1), and phosphorylated Akt (Ser473).

**Figure 10:**
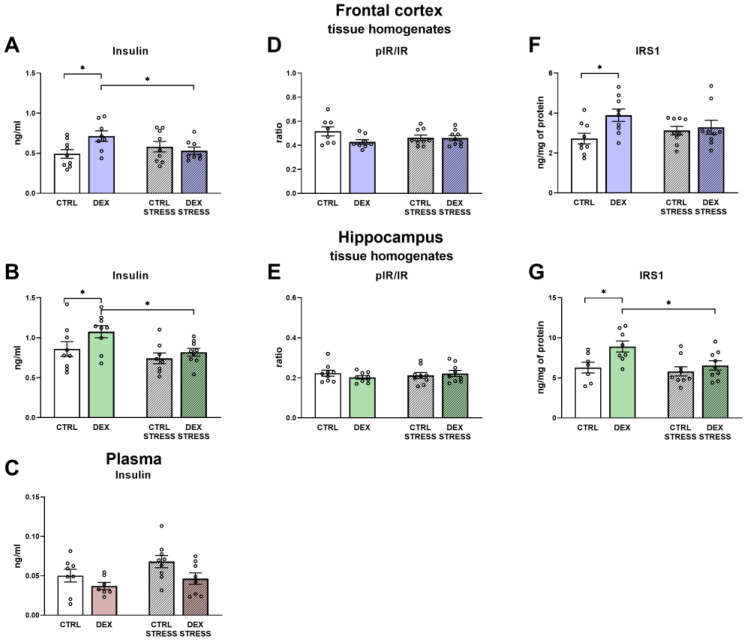
The effects of DEX treatment and acute stress on the levels of insulin, phospho-insulin receptor (pIR/IR), IRS1 in the tissue homogenates of the frontal cortex (**A**,**C**,**E**) and the hippocampus (**B**,**D**,**F**) and insulin in the plasma (**G**); proteins levels were determined with ELISA assays; * *p* < 0.05, *n* = 7–9 The results are expressed as the mean ± SEM. Statistics: two-way ANOVA, followed by the Duncan test.

DEX administered in the third trimester of pregnancy resulted in the elevation of insulin levels in both investigated brain areas of adult male rats (DEX × Stress effect F_1,30_ = 5.48; *p* = 0.026 in the frontal cortex; DEX effect F_1,30_ = 5.95; *p* = 0.021 and Stress effect F_1,30_ = 9.11; *p* = 0.005, in the hippocampus) (Figure 10A,B). In the DEX Stress group, such an effect was not observed. To ensure that the observed effects were not the result of insulin transported across the blood-brain barrier (BBB), the plasma insulin level was measured, and no changes were detected (Figure 10C).

Additionally, the calculated ratios of pIR/IR in both brain structures revealed no changes in the activation of the receptor (Figure 10D,E).

As shown in Figure 10F,G, dexamethasone administration caused an increase in the level of IRS1 in both brain areas (DEX effect F_1,32_ = 5.26; *p* = 0.028 for frontal cortex; DEX effect F_1,29_ = 7.18; *p* = 0.012 for hippocampus). Additionally, in the DEX-treated group subjected to stress, IRS1 was reduced in the hippocampus compared to the group without stress (Stress effect F_1,29_ = 5.10; *p* = 0.032).

**Figure 11:**
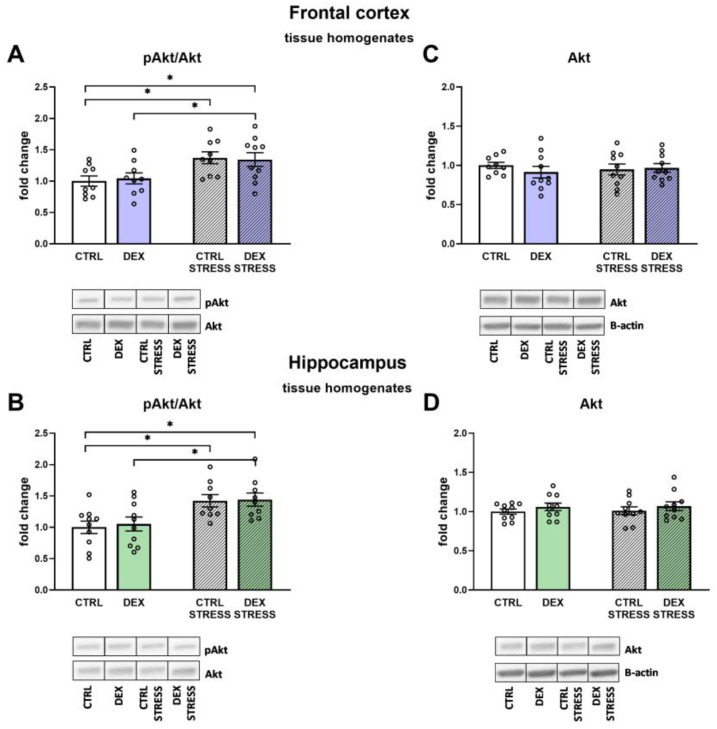
The effects of DEX treatment and acute stress on the levels of phospho-Akt (pAkt/Akt) and total Akt in the tissue homogenates of the frontal cortex (**A**,**C**) and the hippocampus (**B**,**D**); proteins levels were determined with Western blotting analysis; * *p* < 0.05, *n* = 9–10. The results are expressed as the average fold change ± SEM. Statistics: two-way ANOVA, followed by the Duncan test.

In both the frontal cortex and the hippocampus, the effects of stress were observed in studies on protein kinase B (Akt) phosphorylation. In the Control and DEX-treated groups, stress led to an increased level of phosphorylation of the examined kinase (Stress effect F_1,33_ = 12.40; *p* = 0.001 for frontal cortex; Stress effect F_1,34_ = 15.22; *p* = 0.001 for hippocampus, Figure 11A,B) with no changes in total Akt form (Figure 11C,D).

#### 2.2.11. The Effects of Dexamethasone Treatment and Acute Stress on Uncoupling Proteins (UCP2 and UCP4) in the Mitochondria-Enriched Fraction of the Frontal Cortex and Hippocampus

Since it is known that mitochondrial respiratory oxidation coupled with ATP synthesis can be disrupted by UCP proteins, which are transporters present in the mitochondrial inner membrane that mediate a regulated discharge of the proton gradient, we measured UCP2 and UCP4 to exclude this mechanism in disturbances of energy production in our model.

The UCP2 level in the frontal cortex was lowered by prenatal DEX treatment (DEX effect F_1,32_ = 4.368; *p* = 0.045) in both groups in comparison to the Control (Table 1). No changes were detected taking into account UCP2 levels in the hippocampus and UCP4 in either studied brain area (Table 1).

#### 2.2.12. The Effects of Dexamethasone Treatment and Acute Stress on Mitochondrial Fission and Fusion Processes (MFN2, OPA1-L, and OPA1-S Protein Levels) in the Mitochondria-Enriched Fraction of the Frontal Cortex and Hippocampus

One of the causes of the dysregulation of energy production in cells may also lie in the dynamics of mitochondria. The processes behind this concept, including fission and fusion of these organelles, are regulated by a number of mitochondrial proteins, two of which we have selected for further investigation, that play key roles in these processes i.e., MFN2 and OPA1.

No differences in MFN2, OPA1-L, and OPA1-S protein levels were detected in either the frontal cortex or the hippocampus. The calculated OPA1-L/S ratio was reduced in the frontal cortex in the DEX Stress group compared to the Control animals subjected to acute stress sessions (DEX effect F_1,29_ = 10.57; *p* = 0.029) (Table 1).

## 3. Discussion

The animal model of depression used in the present study was positively verified in our lab by demonstrating the behavioral changes characteristic of depression in the male offspring of mothers receiving DEX in the third trimester of pregnancy. The presence of depressive-like behaviors was evidenced by the extended immobility time shown in the FST and anxiety behavior, a symptom common in depression, demonstrated by a decrease in the open arm entries and a reduction in the time spent in open arms in the EPM. Moreover, this model of depression based on DEX prenatal administration was previously behaviorally and pharmacologically verified by other authors [21,22]. Selected administration time and a low dose (0.1 mg/kg) were intended to simulate the conditions of dexamethasone use in the clinic. However, it should be noted that our schedule of treatment differed from that used in the clinic as dexamethasone was administered 1/3 of the gestation period and in humans it is administered in several divided doses.

In the depression model used, we demonstrated the presence of metabolic changes in the brain, which were stronger in the frontal cortex than in the hippocampus and indicated a reduction in the oxidative phosphorylation process. Clear shifts in the intensity of individual metabolic processes occur in the frontal cortex, where observed changes indicate a reduction in the phosphorylation process and probably the Krebs cycle with the simultaneous accumulation of glycolysis products. The reduction in the oxidative phosphorylation process in the frontal cortex of animals receiving DEX in the prenatal period was shown by both the reduction in the level of mitochondrial complexes II, IV, and V and the reduction in mitochondrial respiratory function determined by measuring the rate of oxygen consumption via the respirometry method. A decrease in the level of pyruvate dehydrogenase with a simultaneous increase in the level of pyruvate in the mitochondria-enriched fraction of the frontal cortex indicates that the Krebs cycle is also weakened. This enzyme is responsible for the conversion of pyruvate into acetyl-CoA, which can be included in the Krebs cycle. Since a decrease in the expression of this enzyme was observed without changes in its activity, it suggests a weakening of the connection between glycolysis and the Krebs cycle. As no changes in the expression of key glycolysis enzymes were observed, it appears that the increased levels of pyruvate in the mitochondria-enriched fraction and cytosolic fractions and lactate in the cytosolic fraction are due to a reduction in PDH expression and not an increase in glycolysis. Importantly, the observed changes lead to a reduction in the level of ATP and thus the energy needed for the proper function of nerve cells. In previously studied models of depression based on prenatal stress [23,24] or in Wistar–Kyoto rats [25,26], we also observed metabolic changes in brain structures, but they did not lead to a reduction in ATP levels. Thus, compared to these models, the prenatal DEX depressive model used in the present study results in more pronounced metabolic changes in the frontal cortex, which consequently reduces ATP production. Moreover, in previous models, metabolic changes occurred not under baseline conditions but mainly after the use of additional unfavorable factors applied in adult life. In the case of prenatal administration of DEX, the changes described above in the frontal cortex were already present in the basal condition, while subjecting animals to stress in adulthood mainly increased the levels of pyruvate kinase and phosphofructokinase, which points to an intensification of the glycolysis process.

In contrast to the frontal cortex, in the hippocampus, no changes in the examined metabolic markers, mitochondrial respiratory function, or ATP level in animals prenatally exposed to DEX were observed. When animals receiving prenatal DEX were subjected to acute stress, there was a significant reduction in pyruvate dehydrogenase activity in the hippocampus, which may suggest a weakening of the Krebs cycle due to reduced acetyl-CoA supply. Acute stress also influenced the levels of key glycolysis enzymes (decreased level of phosphofructokinase and increased hexokinase). Thus, in the present model of depression in the frontal cortex, metabolic processes were reduced, and stress in adulthood only slightly modified these changes, while in the case of the hippocampus, prenatal exposure to DEX did not cause long-term metabolic changes but made this structure more sensitive to future adverse action.

Since the main change in the frontal cortex in the depression model used was a reduction in the oxidative phosphorylation process with a simultaneous increase in the level of glycolysis end products, we tried to determine the probable mechanism of these changes in further studies. One of the possible causes of disturbances in brain energy metabolism is the detachment of mitochondrial hexokinase from the outer mitochondrial membrane, which affects the activity of this enzyme and couples cytosolic glycolysis with mitochondrial oxidative phosphorylation [27]. The level of HK in the mitochondria-enriched fraction did not change, but the expression of isoform 1 of the VDAC1, which binds HK to the mitochondrial membrane, was significantly reduced in both examined brain structures. Thus, lowering VDAC1 levels may be one of the causes of DEX-induced metabolic disturbances in the brain, including disrupting the coordination between glycolysis and oxidative phosphorylation. Moreover, proper binding of HK to the mitochondrial membrane, by reducing the production of mitochondrial reactive oxygen species, prevents oxidative damage and the induction of the mitochondrial apoptosis pathway [28]. In turn, the connection of HK with the mitochondrial membrane is enhanced by the phosphatidyl inositol 3-kinase (PI3-K)-Akt signaling pathway, and this survival signal is potentiated by insulin. In fact, it has been shown that the decrease in the insulin receptor in the brain, as seen in schizophrenia, leads to HK mitochondrial detachment [29]. In the present study, we did not observe a decrease in phosphorylated, active insulin receptor levels, but in animals prenatally exposed to DEX, some changes indicative of insulin brain resistance, such as increased insulin levels in the frontal cortex and hippocampus, but not in plasma, and an increase in insulin receptor substrates 1 (IRS1) in the frontal cortex and the hippocampus were present. IRS1 plays a significant role in the induction of signal transduction pathways in insulin action; thus, it is possible that its increased level may also be accompanied by excessive phosphorylation of serine at position 307, which, as a result, may induce insulin resistance and evoke metabolic and synaptic plasticity disturbances [30]. However, we have not assayed the levels of various phosphorylated forms of IRS1, and in addition, there was no reduction in Akt and *p*-Akt levels, which did not confirm the development of insulin resistance in the examined brain structures in animals prenatally exposed to DEX.

The decrease in the oxidative phosphorylation process, shown mainly in the frontal cortex, may result not only from changes in the expression/activity of enzymes involved in this process but also from changes in the dynamics of mitochondria. Mitochondria are very dynamic organelles that, according to the energy demand of the cell, are subject to the process of fusion and fission, and these processes are regulated by a number of mitochondrial proteins [31,32]. The lack of change in the levels of the key fusion proteins mitofusin 2 and optic atrophy 1 (OPA1) in both examined brain structures excludes the participation of this process in decreasing oxidative phosphorylation. Since OPA-1 can be converted from the long form (OPA1-L) to the short form (OPA1-S) and some data indicate that only the long isoform promotes mitochondrial fusion, while the short form enhances fission [33], we also assessed the ratio of these two forms. The reduction in the OPA1-L to OPA1-S ratio in animals in the DEX/stress group suggested that DEX may sensitize the frontal cortex to stress-induced mitochondrial disturbances.

The present results also indicate that the decrease in ATP levels observed in the frontal cortex is not due to uncoupling of oxidative phosphorylation from ATP synthesis. This is demonstrated by the lack of an increase in the level of the major of uncoupling proteins occurring in the brain, that is, UCP2 and UCP4, as well as the lack of intensification of the oxygen consumption rate in the leak state in respirometric examination, that is, under conditions of inactive ATP synthesis in which oxygen flux is maintained mainly to compensate for the proton leak. In contrast, a reduction in the level of UCP2 in the frontal cortex of animals prenatally exposed to DEX, both without and after exposure to acute stress in adulthood, suggests that DEX may reduce proton leakage through the mitochondrial membrane.

The results obtained from the present study indicate that a reduction in oxidative phosphorylation and, as a result, ATP synthesis may result from the reduced transport of lactate from astrocytes to neurons and pyruvate transport into the mitochondria. The transport of lactate to neurons, cells that need a large amount of energy to maintain proper function, is an important mechanism. Most lactate is produced in the process of glycolysis in astrocytes and is delivered to neurons (lactate shuttling), where after conversion to pyruvate, it feeds the process of oxidative phosphorylation [34]. Monocarboxylate transporters play an important role in the lactate shuttling process, and current data show that MCT4 is responsible for the outflow of lactate from astrocytes, while MCT2 is associated with the influx of lactate to neurons [35,36]. In animals prenatally exposed to DEX, we observed a stress-induced reduction in the levels of both of these transporters, e.g., MCT2 and MCT4. The fact that, in animals exposed to DEX, stress reduced both lactate transport to neurons and pyruvate transport to the mitochondria (MPC1) only in the frontal cortex, and not in the hippocampus, suggests that this may be a significant cause of the reduction of oxidative phosphorylation and ATP synthesis in this structure. It should be noted, however, that the reduction of these transporters occurred in animals exposed to DEX, but only after being stressed in adulthood, while the reduction in ATP levels was observed under basic conditions in DEX-treated rats.

**Figure 12:**
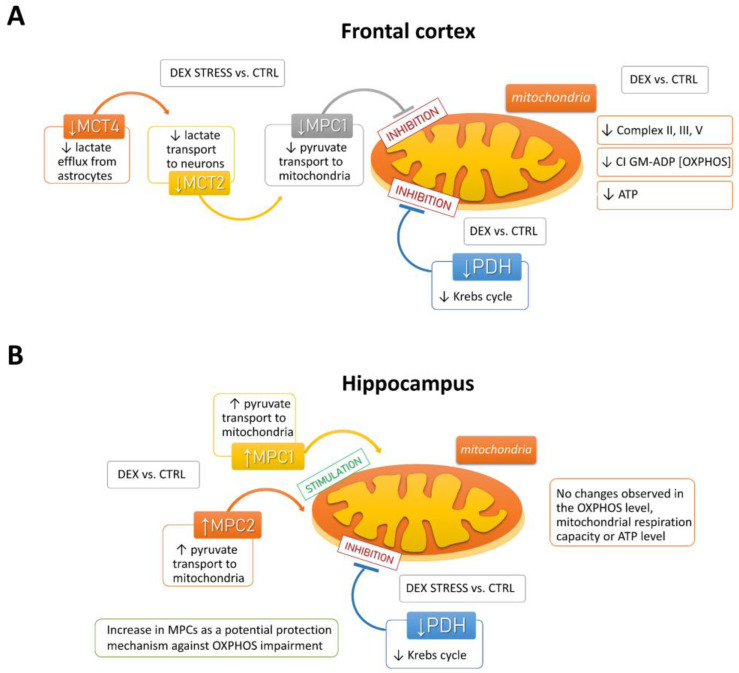
Scheme summarizing the effect of DEX and DEX stress on the dysregulation of energy production in the frontal cortex (**A**) and hippocampus (**B**).

In contrast to the frontal cortex, MPC1 and MPC2 levels increased in the hippocampus in animals exposed to DEX, suggesting that this may be a mechanism that protects this structure from DEX-induced reduction in ATP synthesis (summary scheme of described dependencies is shown in Figure 12). Moreover, in the hippocampus of animals prenatally exposed to DEX, an increase in lactate G-protein-coupled hydroxycarboxylic acid receptor 1 (HCAR1; GPR81) levels was also observed. It is now known that lactate acts in the brain not only as a metabolic substrate but also as a signaling molecule by activating the GPR81 receptor and most likely also through the yet unidentified GPCR coupled to the Gs receptor [37,38]. Since L-lactate has been shown to enhance cognitive function (learning and long-term memory) and exert neuroprotective effects in models of ischemia and exitotoxicity [39,40,41]), the intensification of GPR81 levels in the hippocampus seems to be, as in the case of MPC1 and MPC2 transporters, a favorable change.

In conclusion, in a model of depression based on prenatal exposure of animals to DEX, pronounced metabolic disturbances in the brain of adult animals occurred in the frontal cortex but not in the hippocampus. The changes demonstrated in the frontal cortex indicate a reduction in the oxidative phosphorylation process, resulting in reduced ATP synthesis and possibly a weakening of the Krebs cycle with the simultaneous accumulation of glycolysis products. It seems that these changes result mainly from a decrease in PDH expression, the impairment of lactate transport to neurons and pyruvate to the mitochondria-enriched fraction, and the development of insulin resistance cannot be ruled out. Contrary to the frontal cortex, there was no decrease in ATP synthesis in the hippocampus, which may be due to the intensification of compensatory and protective mechanisms in this structure, such as an increase in the level of mitochondrial pyruvate transporters or lactate GPR81 receptors. However, these protective changes were no longer present in adult stressed animals.

### Future Research

In future studies, the metabolic nature of depression induced by prenatal stress factors needs to be further investigated. We look forward to broadening the knowledge of the mechanisms causing bioenergetic changes in studying the neuropeptides with a multidirectional activity spectrum, namely, the orexin/hypocretin system. The expression of orexins is strongly regulated by glucocorticoids and the activity of orexin neurons rapidly changes in response to stress. Studying the role of orexin signaling in stress reactivity, as well as describing how dysregulation of this system can result in depressive phenotype will expand current research. Nowadays, due to the great interest in the therapeutic potential of pharmacological compounds that target orexin pathways for the treatment of psychiatric disorders such knowledge will be especially valuable.

## 4. Materials and Methods

### 4.1. Animals and Treatment

All experiments were conducted to minimize animal suffering and to reduce the number of animals used (3R policy).

Sprague-Dawley rats were purchased from Charles River Laboratories (Sulzfeld, Germany) and kept under standard conditions: room temperature of 22 ± 2 °C on a 12 h light/dark cycle, with food and water available ad libitum.

After quarantine, vaginal smears were collected from each female on consecutive days to provide information on the phase of the estrus cycle. When the proestrus phase was recognized based on the smear examination, dams were put in cages with breeding males overnight, and the next morning tested for the presence of sperm. Females with sperm cells detected were randomly assigned to the test and control groups and relocated to separate cages. The experimental group (*n* = 6) received the synthetic glucocorticoid, dexamethasone 21-phosphate disodium salt (DEX, Sigma-Aldrich, Saint Louis, MO, United States, Cat# D1159) treatment, which was administered subcutaneously to pregnant females at a concentration of 0.13 mg/kg/mL (equal to 0.1 mg/kg dexamethasone) dissolved in 0.9% saline starting from the 14th day of pregnancy until delivery. Accordingly, saline (0.9%) was administered to the control group’s females (*n* = 6). Male offspring were kept with mothers until weaning (3 weeks). After that time, they were mixed within the specific groups (DEX, *n* = 30 or Control *n* = 30) and housed in groups of three to five animals per cage until 10 weeks of age.

Then, behavioral tests, including FST and the EPM test, were conducted.

Two hours before being sacrificed, subgroups of the DEX-treated (*n* = 10) and Control animals (*n* = 10) were additionally subjected to a single session of acute immobilization stress. For one hour, animals were immobilized in tight cages and then returned to their home cages. One hour later, they were decapitated. A schematic diagram of the experiment is presented in Figure 1.

### 4.2. Behavioral Tests

#### 4.2.1. Elevated Plus Maze Test (EPM)

The anxiety-like behavior in the Sprague-Dawley rats involved in this study was assessed using an EPM made of wood consisting of two open arms elevated 50 cm above the floor. The apparatus was illuminated from beneath with only dim light (15 W). Rats were allowed to adapt to the experimental room for 1 h before the experiments. At the beginning of the experiment, each rat was placed in the center of the maze facing an open arm [42]. The percent of time spent in the open arm ([time spent in the open entries]/[time spent in both arms] × 100) and the percent of the open arm entries ([open entries]/[total entries] × 100) were estimated during the 5 min experiment.

#### 4.2.2. Forced Swim Test (FST)

The FST, developed by Porsolt et al. [43,44], is the most widely used tool for evaluating depression-like states in rodents and assessing antidepressant drug efficiency [45]. The FST is based on the observation that rodents, following initial escape-oriented swimming, develop an immobile posture when placed in an inescapable cylinder of water. The test was conducted for 2 constitutive days. On the first day (pretest), each animal was placed in a water-filled cylinder with 35 cm water at 23–25 °C for 15 min. On the second day, the test was performed under the same conditions, and the immobility time was measured for 5 min.

### 4.3. Tissue Preparation

The rats were sacrificed under nonstressed conditions by rapid decapitation (between 9 a.m. and 12 p.m.). After that, trunk blood samples were collected into Falcon tubes containing EDTA and centrifuged (3000 rpm, 20 min, 4 °C). The separated plasma was transferred into new tubes and frozen at −20 °C. The rat brains were removed, and the frontal cortices and hippocampi were immediately dissected on ice-cold glass plates and further processed depending on their intended purpose. Samples collected for mitochondrial respiratory experiments (Oxygraph-2K study) required freshly isolated brain tissues (details of sample preparation in Section 4.7). Samples for luminescent assessment of ATP content were homogenized immediately after tissue dissection and then frozen and stored at −80 °C until use (details of sample preparation in Section 4.8). For the remaining biochemical analyses, tissue samples were frozen and stored at −80 °C until use.

### 4.4. Sample Preparation

#### 4.4.1. Isolation of the Mitochondria-Enriched and Cytosolic Fractions

To assess the amount and activity of some mitochondrial enzymes, the mitochondria-enriched membrane fraction was isolated from the frontal cortices and hippocampi according to Wernicke et al. [46]. Briefly, brain tissues were homogenized in ice-cold homogenization buffer (5 mol/L HEPES/NaOH, pH 7.4, 320 mmol/L sucrose, and 1 mmol/L Na_+_/EDTA) with the addition of 0.5% protease inhibitor cocktail (Protein Inhibitor Cocktail, Sigma-Aldrich, Saint Louis, MO, United States, Cat# P8340) using a Teflon-glass homogenizer. Then, the samples were centrifuged (1300× *g*, 4 min, 4 °C), and the supernatant was collected. The residual pellet was rinsed with homogenization buffer and centrifuged again (1500× *g*, 4 min, 4 °C) to enhance mitochondrial yield. Finally, the combined supernatants were centrifuged (17,000× *g*, 12 min, 4 °C). The mitochondria-containing pellets and supernatant (cytosolic fraction) were frozen and stored at −80 °C until use.

#### 4.4.2. Tissue Homogenate Preparation

Tissue samples were homogenized in 2 mL tubes filled with a buffer appropriate for the specific method to be used using Tissue Lyser II (Qiagen Inc., Valencia, CA, USA) for 5 min at 30 Hz. Then, the samples were shaken for 20 min and finally centrifuged for 20 min at 14,000 rpm. Supernatants were collected, frozen, and stored at −80 °C until use.

### 4.5. Protein Concentration Assessment

The protein level in the tissue homogenates as well as in mitochondria-enriched and cytosolic fractions was determined with the BCA method [47] using the PierceTM BCA Protein Assay Kit (Thermo Fisher Scientific, Waltham, MA, USA).

### 4.6. Western Blotting Assay

The frontal cortex and hippocampus samples were subjected to Western blotting analysis and homogenized in 2% SDS, while the samples of the isolated mitochondria-enriched membrane fraction were lysed in PBS containing 1% Triton X-100 and 0.1% SDS, enriched with phosphate and protease inhibitors (Thermo Fisher Scientific, Waltham, MA, USA, Cat# 78440). The total protein concentration was normalized to 3 μg/µL. Twenty micrograms of total protein mixed with loading buffer (Bio-Rad, CA, Hercules, CA, USA, Cat# 1610747) was boiled at 95 °C for 5 min (homogenate samples) or incubated at 37 °C for 5 min (mitochondria-enriched fraction samples) and proceeded according to a standard Western blotting protocol. The separation of proteins was carried out using Criterion™ TGX™ Precast Midi Protein Gels (Bio-Rad, Hercules, CA, USA) for 1 h under a constant voltage of 150 V, followed by semidry transfer to PVDF membranes (Sigma-Aldrich, Saint Louis, MO, USA). The membranes were then cut to allow simultaneous incubation with various antibodies and blocked with 5% skim milk in Tris-buffered saline (TBS) with 0.05% Tween 20 (Sigma-Aldrich, Saint Louis, MO, USA) for 1 h at room temperature. Primary antibodies against the following proteins were then added and incubated with the membranes at 4 °C overnight: OXPHOS–Total OXPHOS Rodent WB Antibody Cocktail (Abcam, Cambridge, UK, Cat# MS 604-300); Hexokinase-1 (HK1, Proteintech, Wuhan, Hubei, China, Cat# 19662-1-AP); Monocarboxylate Transporter 2 (MCT2, Bioassay Technology Laboratory, Jiaxing, Zhejiang, China, Cat# BT-1P05521); Monocarboxylate Transporter 4 (MCT4, Gene Tex, Alton Pkwy Irvine, CA, USA, Cat# GTX87926); Mitochondrial Pyruvate Carrier 1 (MPC1, Cell Signaling Technology, Danvers, MA, USA, Cat# 14462); Mitochondrial Pyruvate Carrier 2 (MPC2, Cell Signaling Technology, Danvers, MA, USA, Cat# 46141); Hydroxycarboxylic acid receptor 1 (HCAR1, GPR81, Sigma-Aldrich, Saint Louis, MO, USA, Cat# SAB-1300790); Protein Kinase B (Akt, Cell Signaling Technology, Danvers, MA, USA, Cat# 9272); Phosphorylated Protein Kinase B (pAkt, Cell Signaling Technology, Danvers, MA, USA, Cat# 4060); Mitofusin-2 (MFN2, Abcam, Cambridge, UK, Cat# ab56889); Uncoupling protein-4 (UCP4, Thermo Fisher Scientific, Waltham, MA, USA, Cat# PA5-69265); Dynamin-like 120 kDa protein (OPA1, Abcam, Cambridge, UK, Cat# ab42364). The next day, the membranes were washed four times with TBS with 0.1% Tween 20 (TBS-T) for 10 min and incubated with HRP peroxidase-conjugated appropriate secondary antibody: horse anti-mouse or goat anti-rabbit IgG HRP peroxidase-conjugated secondary antibody (both Vector Laboratories, Peterborough, UK, Cat# PI-2000-1 and Cat# PI-1000, respectively) for 1 h at room temperature. After an additional four washes in TBS-T for 10 min, targeted protein bands were detected using BM Chemiluminescence Western Blotting Substrate (POD) (Roche, Mannheim, Germany, Cat# 11500708001) and visualized using a luminescent image analyzer with a Fujifilm LAS-1000 System (Fujifilm, Tokyo, Japan). Relative levels of protein concentration were assessed via densitometry measurements conducted using Fujifilm Multi Gauge software (Fujifilm, Tokyo, Japan). In some cases, the membranes were stripped using stripping buffer containing 100 mL of Tris-HCl (pH = 6.7), 2% SDS, and 700 µL of 2-mercaptoethanol (all from Sigma-Aldrich, Saint Louis, MO, USA) for 0.5 h at 50 °C, washed 3 times for 10 min each in TBS-T, blocked, and reprobed with the appropriate primary antibody. As an internal loading control, anti-β-actin (Sigma-Aldrich, Saint Louis, MO, USA, Cat# A5441) or anti-vinculin (Sigma-Aldrich, Saint Louis, MO, USA, Cat# V9264) antibodies were used. The membranes are addressed in the Supplementary Materials.

### 4.7. Mitochondrial Respiratory Function

Monitoring of mitochondrial respiratory function was conducted using an Oxygraph-2k (Oroboros Instruments, Innsbruck, Austria). For this purpose, frontal cortices and hippocampi were dissected from rats and placed in tubes containing ice-cold homogenization buffer (0.25 M sucrose, 50 mM KCl, 5 mM EDTA, 1 mM sodium pyrophosphate, 5 mM MgCl_2_ (pH 7.4); Sigma-Aldrich, Saint Louis, MO, USA) with freshly added protease inhibitor cocktail (Sigma-Aldrich, Saint Louis, MO, USA, Cat# P8340). To preserve the functionality of mitochondria, brain structures were homogenized ten times using a Teflon-glass homogenizer on ice. Then, they were centrifuged (1300 rpm, 10 min, 4 °C), and the supernatants were collected (SN1). The residual pellets were gently resuspended in four volumes of homogenization buffer and subjected to a second round of centrifugation under the same conditions. After that, the supernatant (SN2) was collected and combined with SN1. The mixture of supernatants was centrifuged again (9000× *g*, 15 min, 4 °C), and the resultant pellets were resuspended in five volumes of homogenization buffer. The protein concentration was measured with the BCA method, and 300 μg (amount determined based on preliminary experiments) of isolated mitochondria was suspended in MiR05 respiration buffer (EGTA 0.5 mmol/L, MgCl_2_·6H_2_O 3 mmol/L, K-lactobionate 60 mmol/L, taurine 20 mmol/L, KH_2_PO_4_ 10 mmol/L, HEPES 20 mmol/L, sucrose 110 mmol/L, fatty acid-free BSA 1 g/L, pH 7.0 with KOH; Sigma-Aldrich, Saint Louis, MO, USA). A total volume of 2 mL of suspension was used per Oxygraph-2k chamber for each experiment.

The measurements were conducted as previously described [25]. Briefly, mitochondrial respiratory function was assessed in the presence of the substrates glutamate and malate. The protocol included measurements as follows: (1) glutamate (10 mM) and malate (2 mM) without ADP (leak state); (2) respiration in the presence of ADP (2.5 mM; ADP-stimulated state, control of coupled respiration by complex I); (3) addition of succinate (10 mM, CI + CII OXPHOS state); (4) titration of the protonophore carbonylcyanide-4-(trifluoromethoxy)-phenylhydrazone (FCCP)-uncoupled state, optimal for 0.25 μM, which is the noncoupled state at the optimum uncoupler concentration for maximum oxygen flux; (5) addition of rotenone (1 μM) (CII-linked ETS capacity); and (6) antimycin A (2.5 μM; inhibition of complexes I and II, respectively). Inhibition of respiration of uncoupled mitochondria leads to the evaluation of oxygen flux due to oxidative side reactions (residual oxygen consumption, ROX). Data from high-resolution respirometry were analyzed using DatLab 4 software (Oroboros, Innsbruck, Austria) and given as an O2 flux per mass, ROX-corrected, and FCCP-normalized.

### 4.8. Luminescence Assay

The intracellular ATP content was measured in freshly homogenized brain tissue immediately after dissection. The commercially available kit ATPliteTM Luminescence Assay System (Perkin Elmer, Waltham, MA, USA, Cat# 6016943), which measures the emission of light proportional to the intracellular concentration of ATP, was used according to the manufacturer’s instructions. Samples were incubated for 10 min in the dark, and the luminescence was measured (with automatic attenuation set and integration time; Tecan Infinite 200 Pro plate reader, Mannedorf, Switzerland). The concentration of ATP was adjusted for the protein content in each sample and reported as µmol/g of protein.

### 4.9. Enzymatic Activity Assay

The activity of pyruvate dehydrogenase (PDH) was measured in the mitochondria-enriched fraction using a commercially available Pyruvate Dehydrogenase Activity Colorimetric Assay according to the manufacturer’s recommendations (BioVision, Milpitas, CA, USA, Cat# K679-100). The absorbance was then measured in a 96-well assay plate in a plate reader at 37 °C for 90 min, with an interval time of 10 min, λ = 450 nm (Tecan Infinite 200 Pro plate reader, Mannedorf, Switzerland). The activity of PDH was then calculated and displayed as nmol NADH/min/mg of protein.

### 4.10. Colorimetric/Fluorometric Assays

In the cytosolic- and mitochondria-enriched fractions, and lactate and pyruvate levels were assessed using a Lactate Colorimetric/Fluorometric Assay Kit (BioVision, Milpitas, CA, USA, Cat# K607-100) and the Pyruvate Activity Colorimetric/Fluorometric Assay Kit (BioVision, Milpitas, CA, USA, Cat# K609-100), respectively. Fractions were deproteinized with the use of a Deproteinizing Sample Preparation Kit according to the manufacturer’s recommendations (BioVision, Milpitas, CA, USA, Cat# 808-200). Samples were transferred into a 96-well plate and mixed with Reaction Mix. After 30 min of incubation, the level of lactate was determined by colorimetric measurements at λ = 570 nm, while pyruvate was determined via fluorometric measurements (excitation wavelength λ = 535 nm, emission wavelength λ = 590 nm; Tecan Infinite 200 Pro plate reader, Mannedorf, Switzerland). The concentrations of the enzymes were calculated based on the standard curve and presented as ng/mg of protein. Protein level was measured before deproteinization.

### 4.11. Enzyme-Linked Immunosorbent Assay (ELISA)

The levels of phosphofructokinase type L (PFKL), pyruvate kinase (PK) and hexokinase (HK), as well as insulin (INS), insulin receptor (IR), phospho-insulin receptor (pIR), and insulin receptor substrate 1 (IRS1), were measured in cortical and hippocampal homogenates prepared in PBS with protease and phosphatase inhibitors using commercially available ELISA kits according to the instructions provided by the manufacturers (Cat# E2541Ra (PFKL), Cat# E1251Ra (PK) and Cat# E0330Ra (HK) from Bioassay Technology Laboratory (Jiaxing, Zhejiang, China); Cat# EZRMI-13K (INS) from Merck Millipore (Darmstadt, Germany); Cat# ELK2640 (IR) and Cat# ELK8434 (IRS-1) from ELK Biotechnology (Wuhan, Hubei, China); Cat# EIA09498r (pIR) from Wuhan Xinqidi Biological Technology (Wuhan, Hubei, China)). Samples of isolated mitochondria-enriched fractions required additional preparation steps. Briefly, the samples were mixed with PBS buffer enriched in protease inhibitors (Protein Inhibitor Cocktail, Sigma-Aldrich, Saint Louis, MO, USA, Cat# P8340), sonicated with an ultrasonic cell disrupter until the solution turned clear, and centrifuged (5000× *g*, 5 min, 4 °C). The supernatants were used to assess the levels of pyruvate dehydrogenase alpha (PDHα), voltage-dependent anion channel 1 (VDAC1), and uncoupling protein 2 (UCP2) with commercially available kits (ELK Biotechnology, Wuhan, Hubei, China, Cat# ELK2703, Cat# ELK7787, and Cat# ELK6519, respectively).

Moreover, in the plasma samples, ELISA was used for insulin (INS) level measurements (ELK Biotechnology, Wuhan, Hubei, China, Cat# ELK2370).

In each case, all samples along with standards and blanks were transferred to 96-well precoated plates. The concentrations of the analyzed markers were calculated based on the standard curves and subsequently divided by the protein content in a given sample, when appropriate. The final concentration was presented as ng/mg of protein.

### 4.12. Statistical Analysis and Data Visualization

Statistical evaluation was conducted using Statistica 13.3 software (StatSoft, Palo Alto, CA, USA) and one- or two-way analysis of variance (ANOVA) followed by the Duncan post hoc test, when appropriate. Differences were considered significant at *p* < 0.05. The ANOVA results are reported as an F-statistic and its associated degrees of freedom. All graphs were prepared using GraphPad Prism 8 (San Diego, CA, USA).

## Figures and Tables

**Table 1:** The effects of DEX treatment and acute stress on the levels of UCP2, UCP4, MFN2, OPA1-L, OPA1-S and their ratio in the mitochondria-enriched fraction of the frontal cortex and the hippocampus; proteins levels were determined with ELISA or Western blotting analysis; * *p* < 0.05 vs. Control group, # *p* < 0.05 vs. Control Stress group, *n* = 8–9. The results are expressed as the mean ± SEM or average fold change ± SEM. Statistics: two-way ANOVA, followed by the Duncan test.

**UCP2** **(ng/mg of protein)**	Frontal Cortex	25.2 ± 2.66	19.83 ± 1.67 *	21.02 ± 1.04	19.23 ± 0.88 *	** 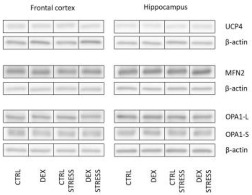 **
	Hippocampus	26.28 ± 0.92	24.57 ± 2.63	27.41 ± 2.83	27.19 ± 1.77	
**UCP4** **(fold change)**	Frontal Cortex	1.00 ± 0.04	1.08 ± 0.05	1.07 ± 0.05	1.05 ± 0.06	
	Hippocampus	1.00 ± 0.05	0.95 ± 0.11	1.01 ± 0.10	1.18 ± 0.14	
**MFN2** **(fold change)**	Frontal Cortex	1.00 ± 0.04	1.01 ± 0.04	1.00 ± 0.05	0.99 ± 0.03	
	Hippocampus	1.00 ± 0.03	1.11 ± 0.08	0.98 ± 0.07	1.02 ± 0.06	
**OPA1-L** **(fold change)**	Frontal Cortex	1.00 ± 0.04	0.89 ± 0.04	0.87 ± 0.04	0.89 ± 0.02	
	Hippocampus	1.00 ± 0.04	0.96 ± 0.06	0.92 ± 0.05	1.05 ± 0.07	
**OPA1-S** **(fold change)**	Frontal Cortex	1.00 ± 0.05	0.98 ± 0.05	0.93 ± 0.08	1.05 ± 0.07	
	Hippocampus	1.00 ± 0.04	0.96 ± 0.04	0.96 ± 0.05	1.06 ± 0.05	
**OPA1-L/S ratio**	Frontal Cortex	1.00 ± 0.03	0.93 ± 0.05	1.09 ± 0.05	0.89 ± 0.04 ^#^	
	Hippocampus	1.00 ± 0.02	1.01 ± 0.04	0.97 ± 0.05	1.00 ± 0.05	

## Data Availability

The data that support the findings of this study are available from the corresponding author upon reasonable request from qualified researchers.

## Supplementary Materials

The following supporting information can be downloaded at: https://www.mdpi.com/article/10.3390/ijms24021156/s1.
