# Supplementary material for: The Effects of Prenatal Dexamethasone Exposure on Brain Metabolic Homeostasis in Adulthood: Implications for Depression
Source: Int J Mol Sci. 2023 Jan 6;24(2):1156. doi: 10.3390/ijms24021156 (PMC9866429; doi:10.3390/ijms24021156)
Supplement: Supplementary file 1 [file ijms-24-01156-s001.zip › ijms-2026309-SI.pdf]

Supplementary materials

Western blot membranes

OXPHOS

Frontal cortex

membrane 1 and membrane 2

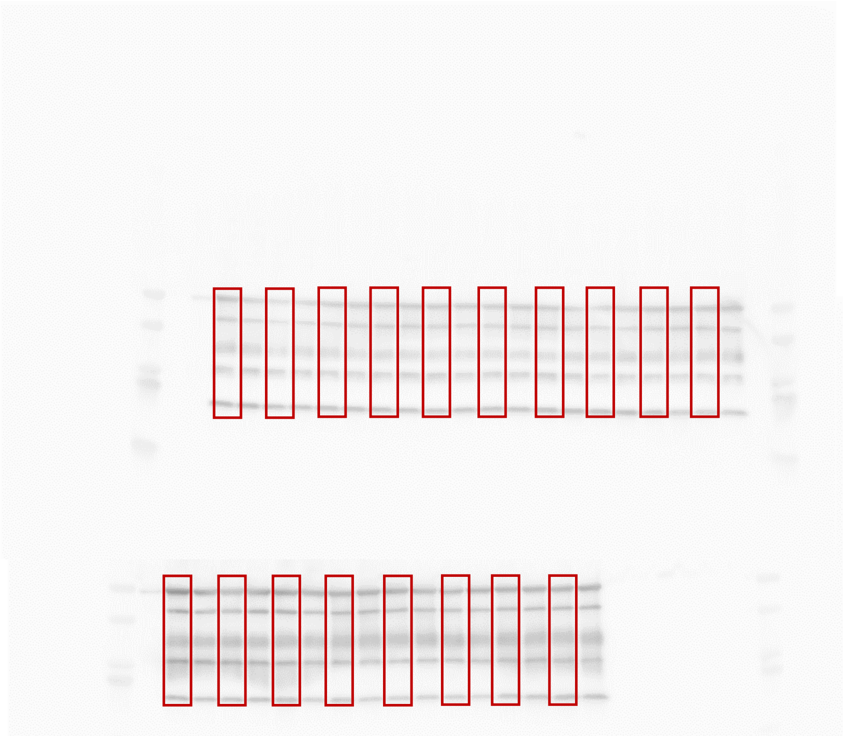

Samples from the left side - membrane 1:
CTRL, DEX, CTRL, DEX, CTRL, DEX, CTRL, DEX, CTRL, DEX.

Samples from the left side - membrane 2:
CTRL, DEX, CTRL, DEX, CTRL, DEX, CTRL, DEX.

The results of the experiment are shown in Figure 3.

β-actin as loading control to OXPHOS

Frontal cortex

membrane 1 and membrane 2

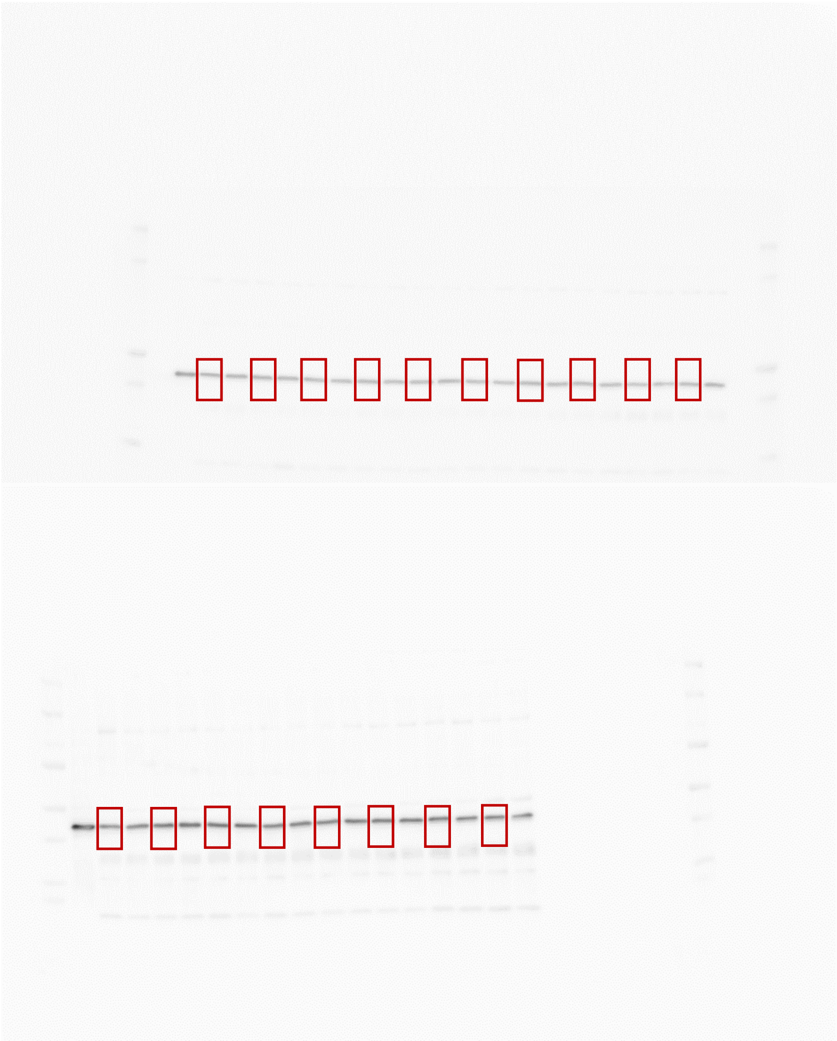

OXPHOS

Hippocampus

membrane 1 and membrane 2

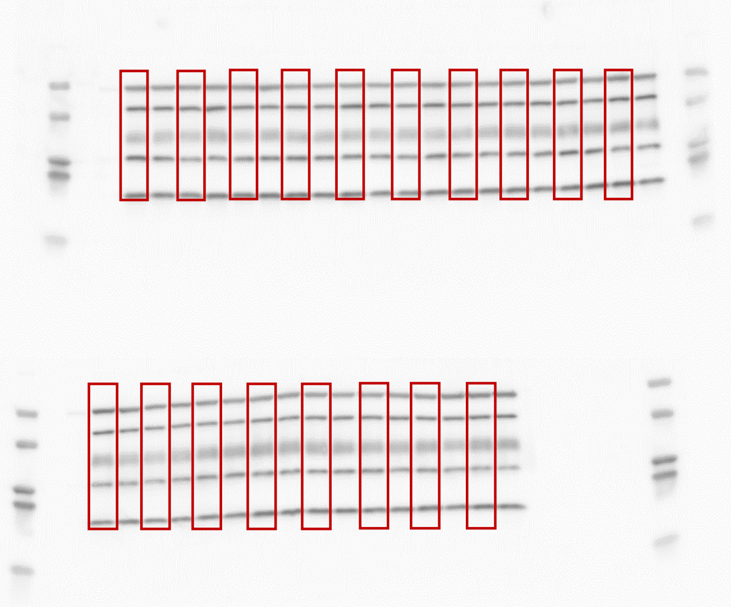

Samples from the left side - membrane 1:
CTRL, DEX, CTRL, DEX, CTRL, DEX, CTRL, DEX, CTRL, DEX.

Samples from the left side - membrane 2:
CTRL, DEX, CTRL, DEX, CTRL, DEX, CTRL, DEX.

The results of the experiment are shown in Figure 3.

β-actin as loading control to OXPHOS

Hippocampus

membrane 1 and membrane 2

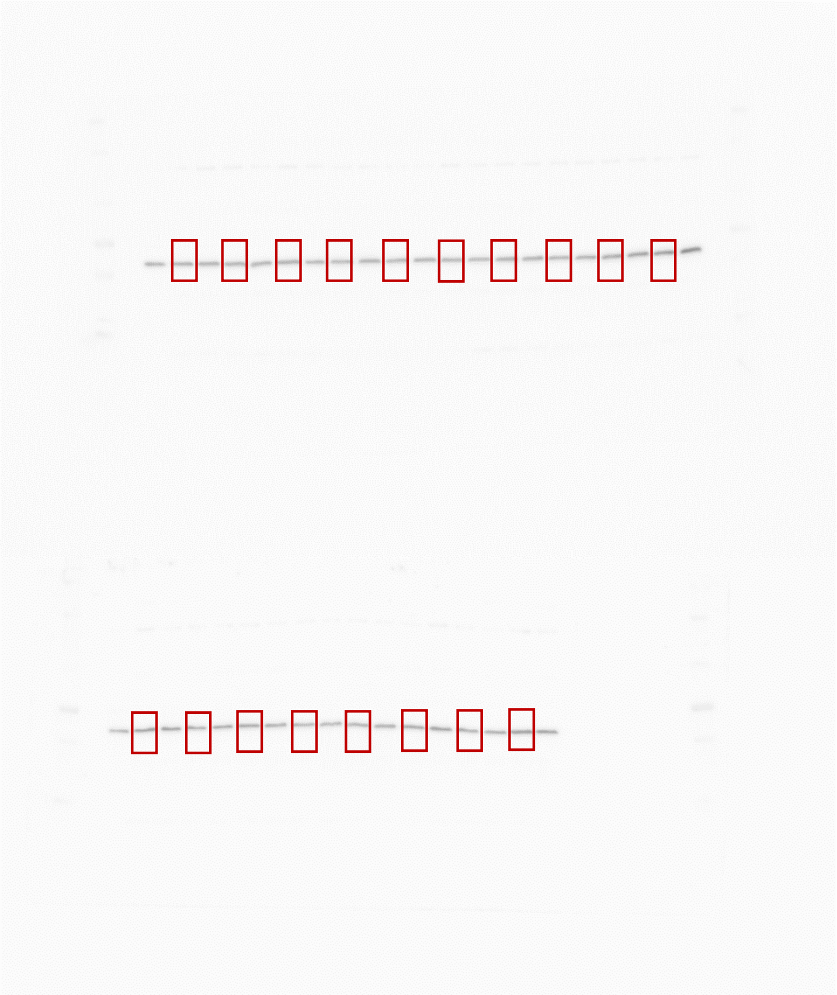

Hexokinase 1

Frontal cortex

membrane 1 and membrane 2

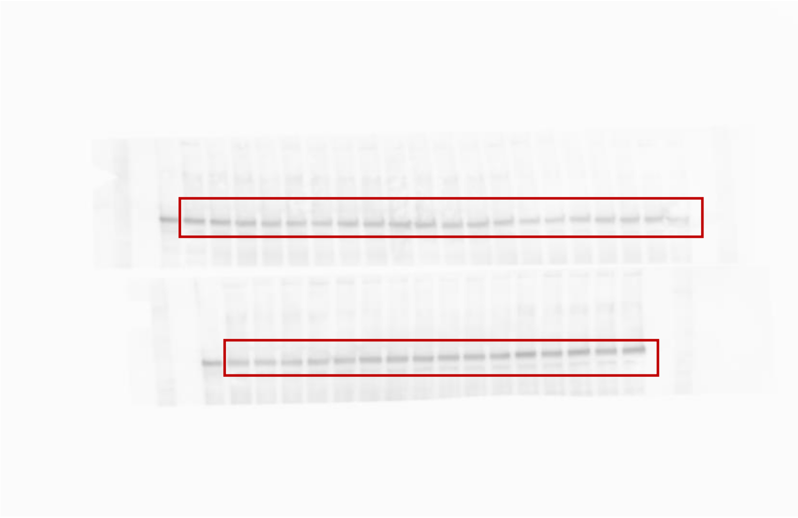

Samples from the left side - membrane 1:
CTRL, CTRL + STRESS, DEX, DEX + STRESS, CTRL, CTRL + STRESS, DEX, DEX + STRESS, CTRL, CTRL + STRESS, DEX, DEX + STRESS, CTRL, CTRL + STRESS, DEX, DEX + STRESS, CTRL, CTRL + STRESS, DEX, DEX + STRESS.

Samples from the left side - membrane 2:
CTRL, CTRL + STRESS, DEX, DEX + STRESS, CTRL, CTRL + STRESS, DEX, DEX + STRESS, CTRL, CTRL + STRESS, DEX, DEX + STRESS, CTRL, CTRL + STRESS, DEX, DEX + STRESS.

The results of the experiment are shown in Figure 7.

β-actin as loading control to Hexokinase 1

Frontal cortex

membrane 1 and membrane 2

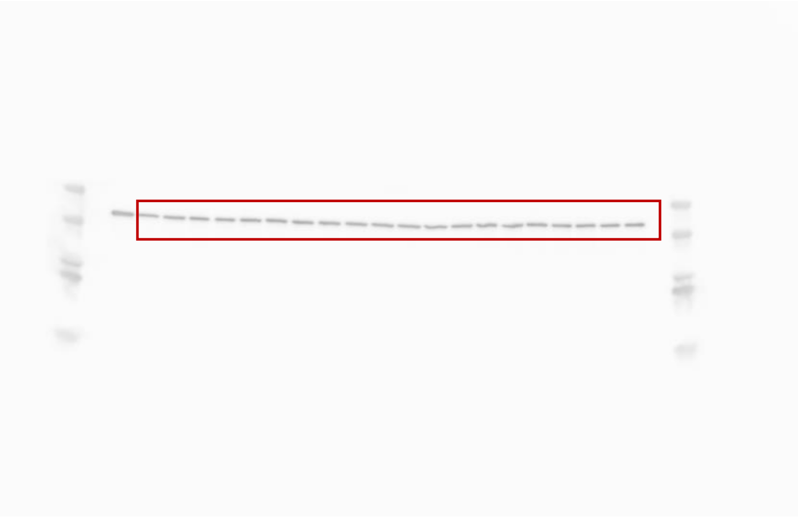

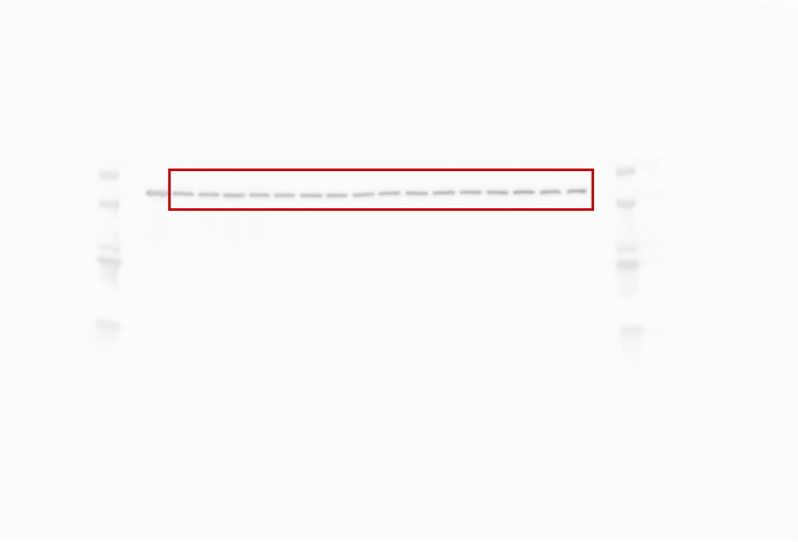

Hexokinase 1

Hippocampus

membrane 1 and membrane 2

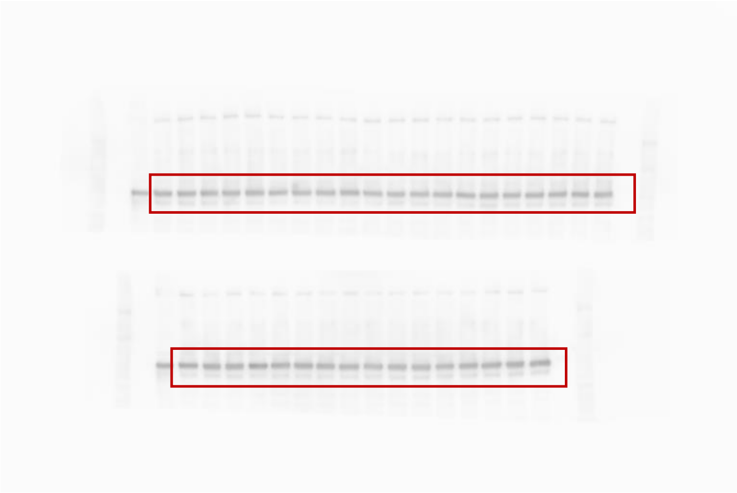

Samples from the left side - membrane 1:
CTRL, CTRL + STRESS, DEX, DEX + STRESS, CTRL, CTRL + STRESS, DEX, DEX + STRESS, CTRL, CTRL + STRESS, DEX, DEX + STRESS, CTRL, CTRL + STRESS, DEX, DEX + STRESS, CTRL, CTRL + STRESS, DEX, DEX + STRESS.

Samples from the left side - membrane 2:
CTRL, CTRL + STRESS, DEX, DEX + STRESS, CTRL, CTRL + STRESS, DEX, DEX + STRESS, CTRL, CTRL + STRESS, DEX, DEX + STRESS, CTRL, CTRL + STRESS, DEX, DEX + STRESS.

The results of the experiment are shown in Figure 7.

β-actin as loading control to Hexokinase 1

Hippocampus

membrane 1 and membrane 2

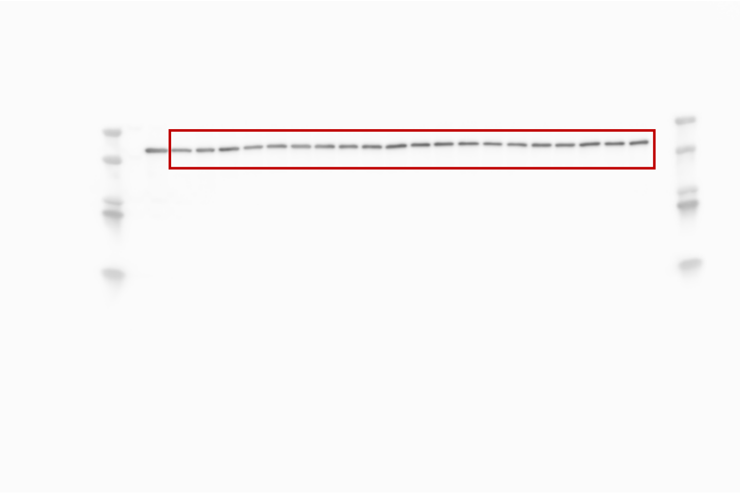

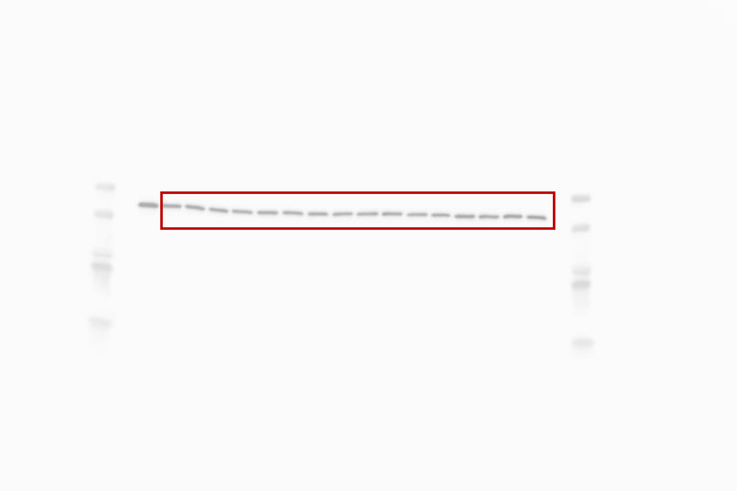

MCT-2

Frontal cortex

membrane 1 and membrane 2

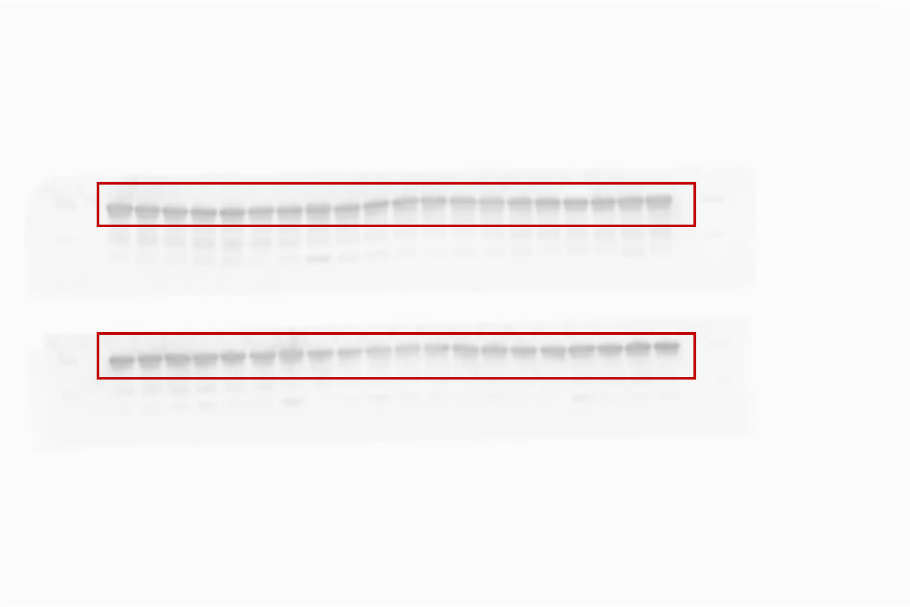

Samples from the left side - membrane 1 and 2:
CTRL, CTRL + STRESS, DEX, DEX + STRESS, CTRL, CTRL + STRESS, DEX, DEX + STRESS, CTRL, CTRL + STRESS, DEX, DEX + STRESS, CTRL, CTRL + STRESS, DEX, DEX + STRESS, CTRL, CTRL + STRESS, DEX, DEX + STRESS.

The results of the experiment are shown in Figure 8.

Vinculin as loading control to MCT-2

Frontal cortex

membrane 1 and membrane 2

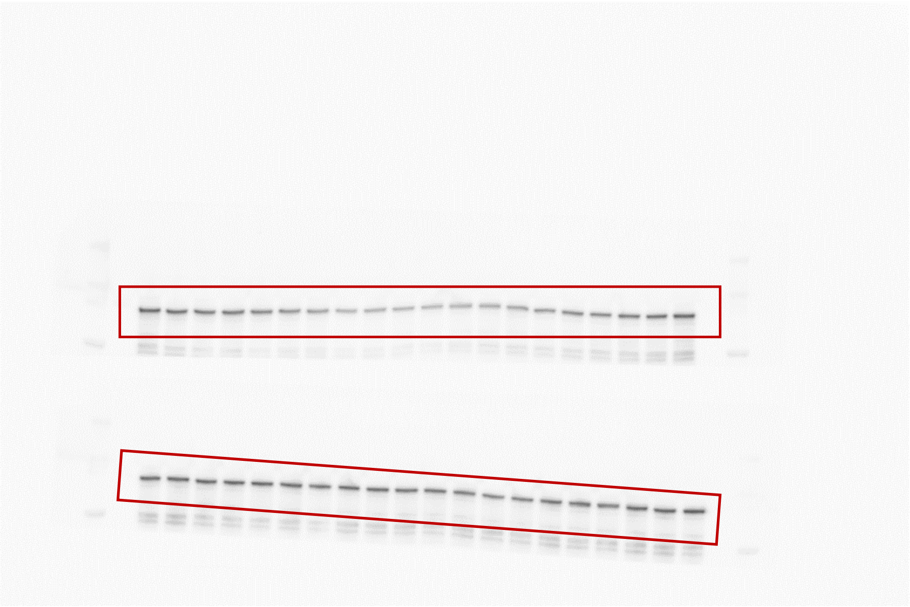

MCT-2

Hippocampus

membrane 1 and membrane 2

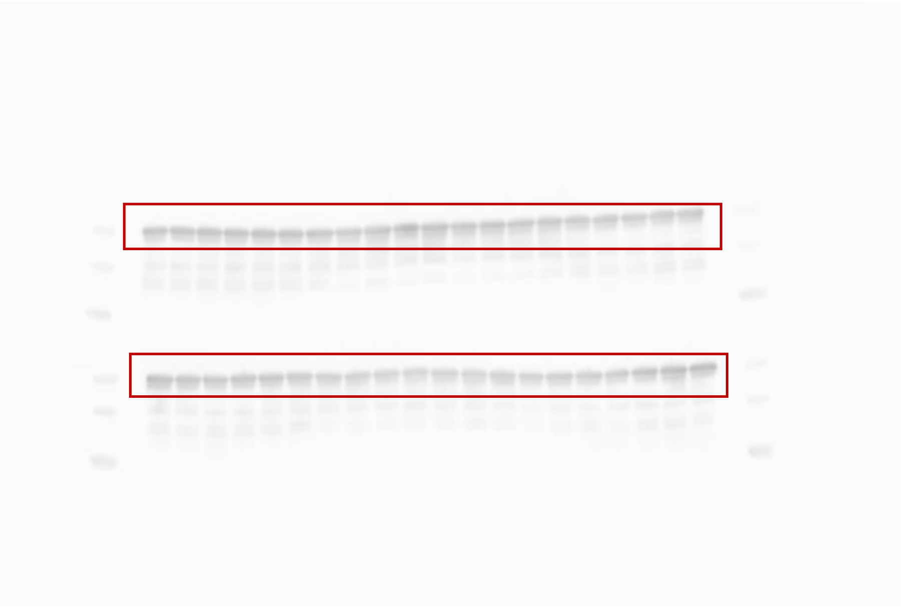

Vinculin as loading control to MCT-2

Hippocampus

membrane 1 and membrane 2

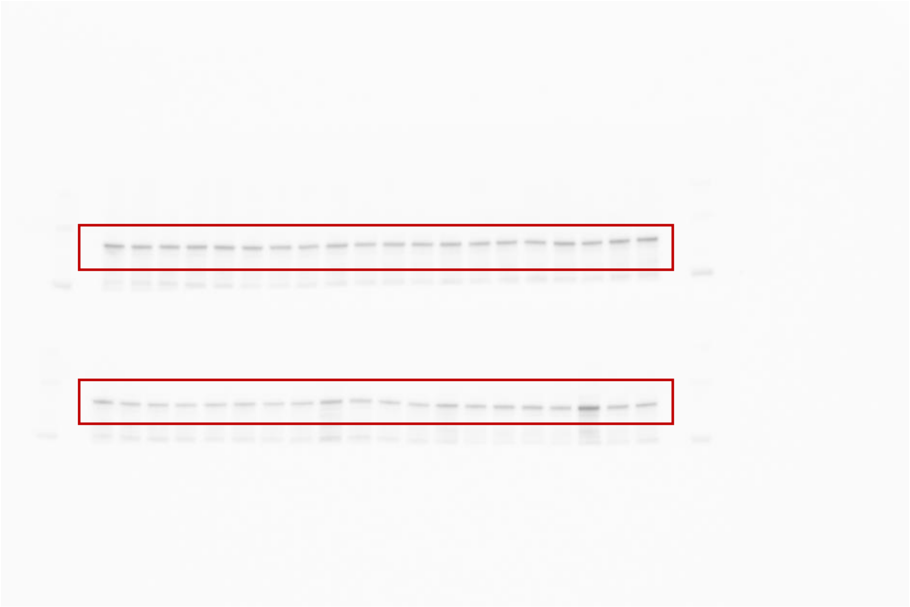

Samples from the left side - membrane 1 and 2:
CTRL, CTRL + STRESS, DEX, DEX + STRESS, CTRL, CTRL + STRESS, DEX, DEX + STRESS, CTRL, CTRL + STRESS, DEX, DEX + STRESS, CTRL, CTRL + STRESS, DEX, DEX + STRESS, CTRL, CTRL + STRESS, DEX, DEX + STRESS.

The results of the experiment are shown in Figure 8.

MCT-4

Frontal cortex

membrane 1 and membrane 2

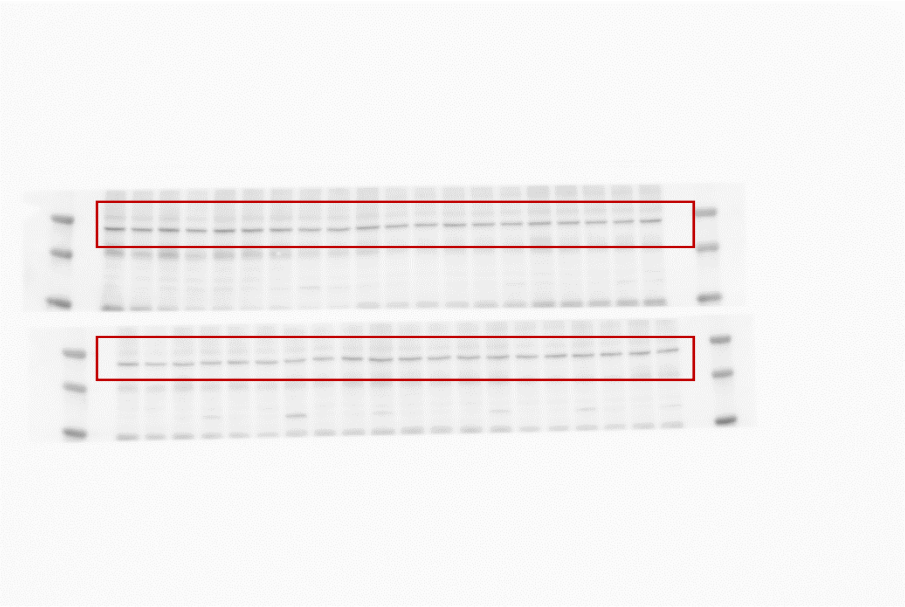

Vinculin as loading control to MCT-4

Frontal cortex

membrane 1 and membrane 2

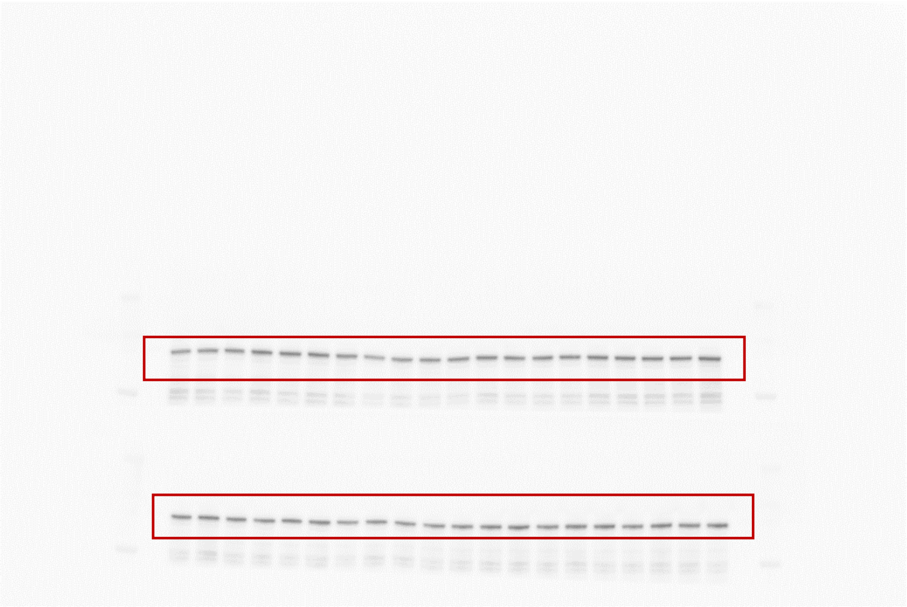

Samples from the left side - membrane 1 and 2:
CTRL, CTRL + STRESS, DEX, DEX + STRESS, CTRL, CTRL + STRESS, DEX, DEX + STRESS, CTRL, CTRL + STRESS, DEX, DEX + STRESS, CTRL, CTRL + STRESS, DEX, DEX + STRESS, CTRL, CTRL + STRESS, DEX, DEX + STRESS.

.

The results of the experiment are shown in Figure 8.

MCT-4

Hippocampus

membrane 1 and membrane 2

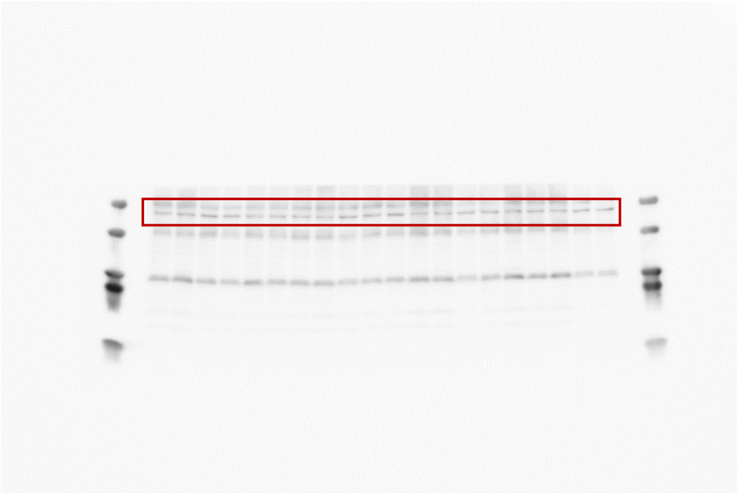

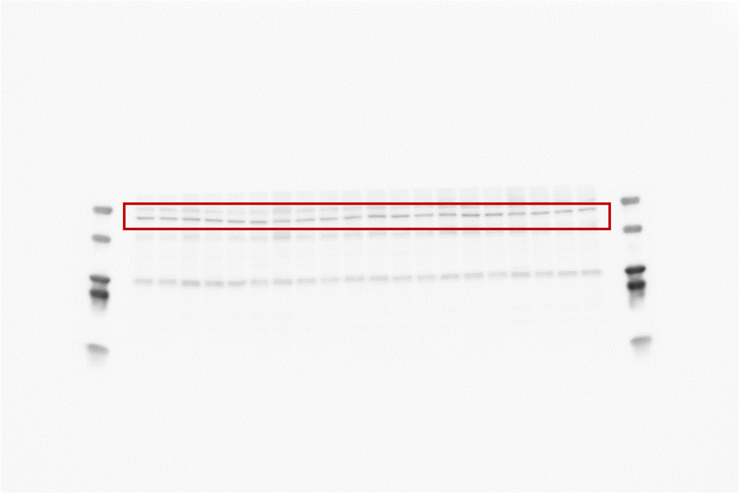

Vinculin as loading control to MCT-4

Hippocampus

membrane 1 and membrane 2

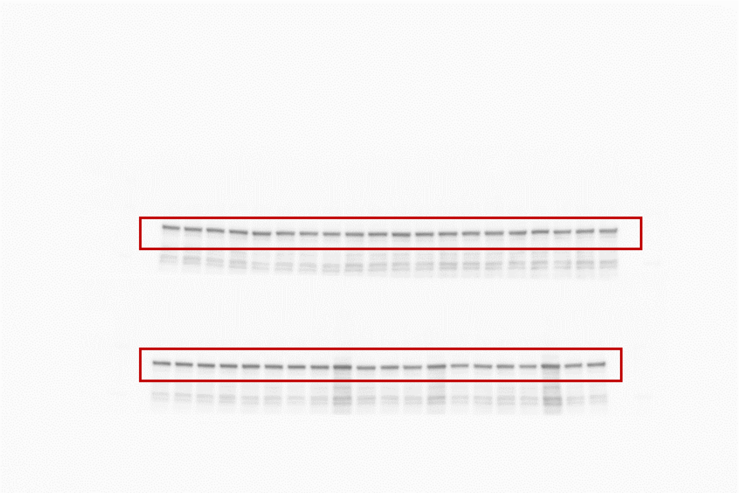

Samples from the left side - membrane 1 and 2:
CTRL, CTRL + STRESS, DEX, DEX + STRESS, CTRL, CTRL + STRESS, DEX, DEX + STRESS, CTRL, CTRL + STRESS, DEX, DEX + STRESS, CTRL, CTRL + STRESS, DEX, DEX + STRESS, CTRL, CTRL + STRESS, DEX, DEX + STRESS.

The results of the experiment are shown in Figure 8.

MPC-1

Frontal cortex

membrane 1 and membrane 2

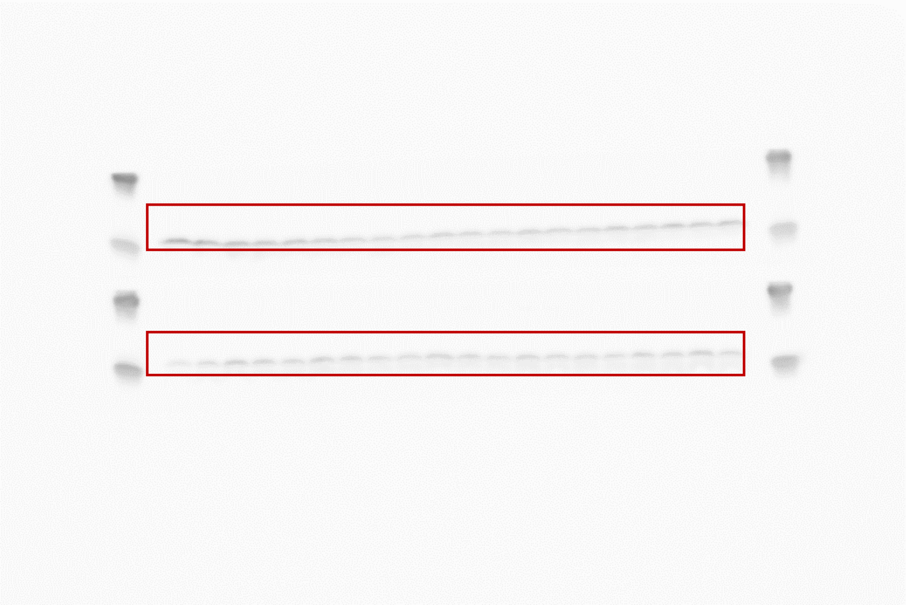

Samples from the left side - membrane 1 and 2:
CTRL, CTRL + STRESS, DEX, DEX + STRESS, CTRL, CTRL + STRESS, DEX, DEX + STRESS, CTRL, CTRL + STRESS, DEX, DEX + STRESS, CTRL, CTRL + STRESS, DEX, DEX + STRESS, CTRL, CTRL + STRESS, DEX, DEX + STRESS.

The results of the experiment are shown in Figure 8.

Vinculin as loading control to MPC-1

Frontal cortex

membrane 1 and membrane 2

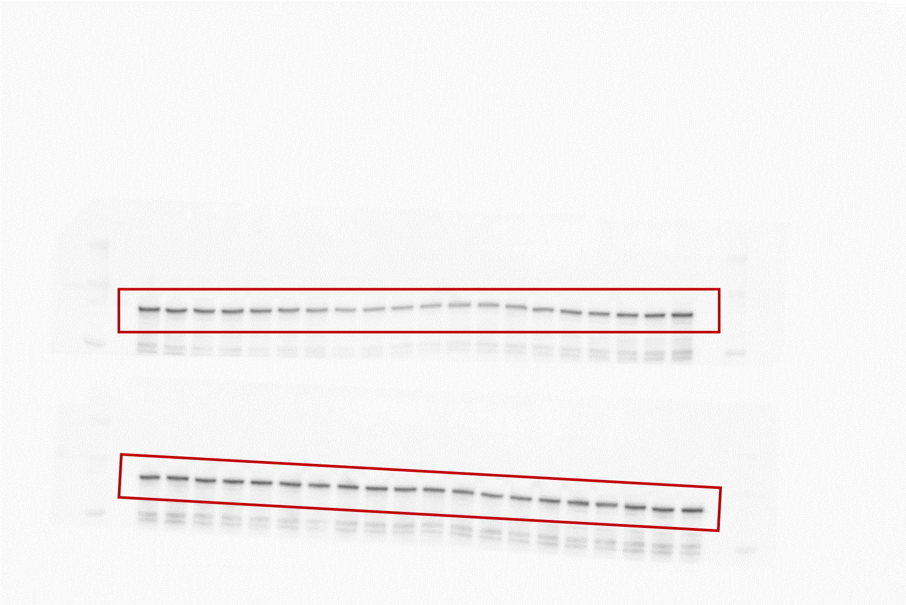

MPC-1

Hippocampus

membrane 1 and membrane 2

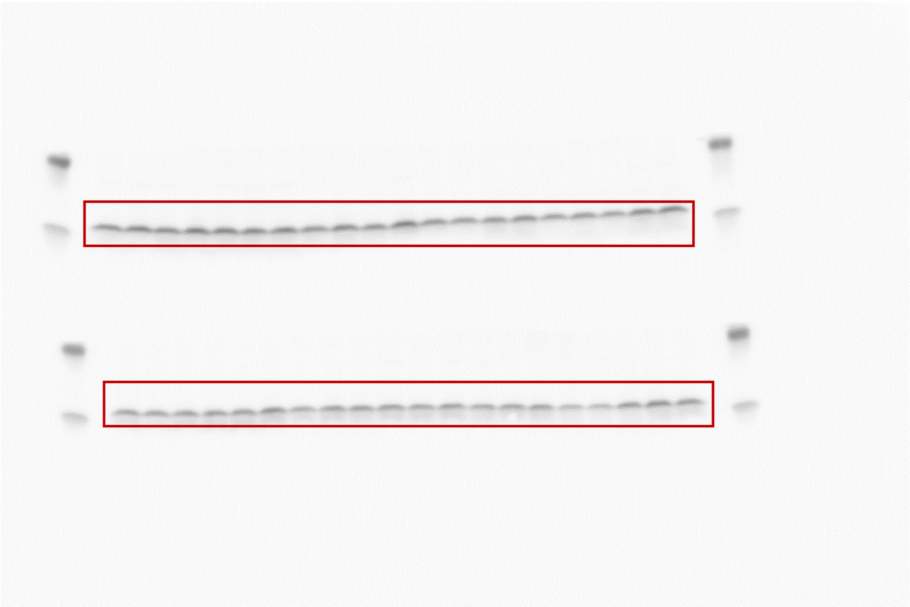

Samples from the left side - membrane 1 and 2:
CTRL, CTRL + STRESS, DEX, DEX + STRESS, CTRL, CTRL + STRESS, DEX, DEX + STRESS, CTRL, CTRL + STRESS, DEX, DEX + STRESS, CTRL, CTRL + STRESS, DEX, DEX + STRESS, CTRL, CTRL + STRESS, DEX, DEX + STRESS.

The results of the experiment are shown in Figure 8.

Vinculin as loading control to MPC-1

Hippocampus

membrane 1 and membrane 2

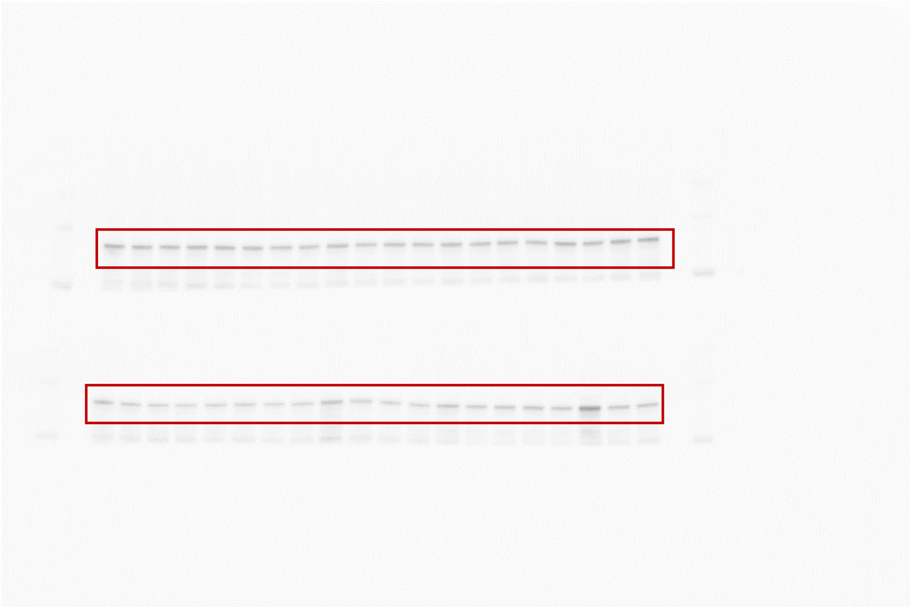

MPC-2

Frontal cortex

membrane 1 and membrane 2

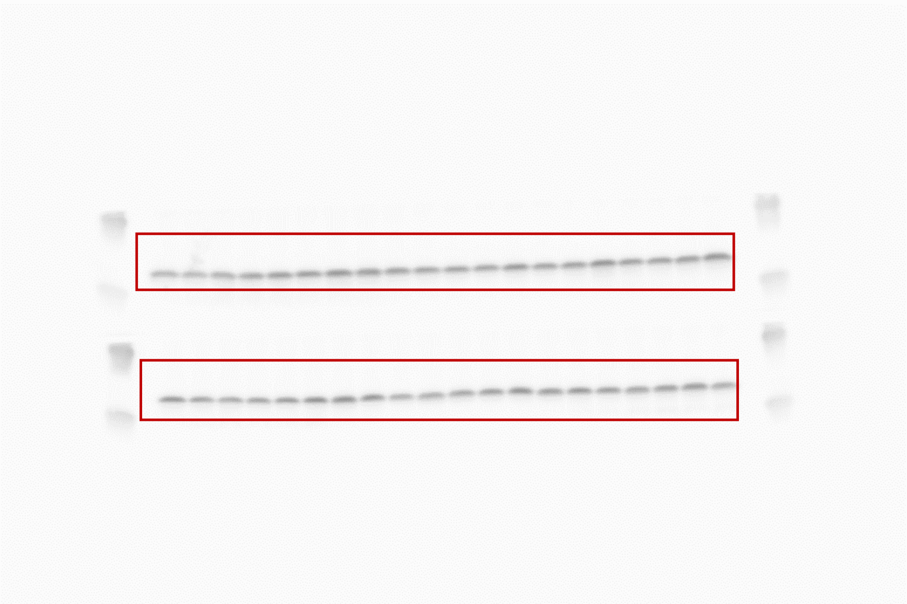

Samples from the left side - membrane 1 and 2:
CTRL, CTRL + STRESS, DEX, DEX + STRESS, CTRL, CTRL + STRESS, DEX, DEX + STRESS, CTRL, CTRL + STRESS, DEX, DEX + STRESS, CTRL, CTRL + STRESS, DEX, DEX + STRESS, CTRL, CTRL + STRESS, DEX, DEX + STRESS.

The results of the experiment are shown in Figure 8.

Vinculin as loading control to MPC-2

Frontal cortex

membrane 1 and membrane 2

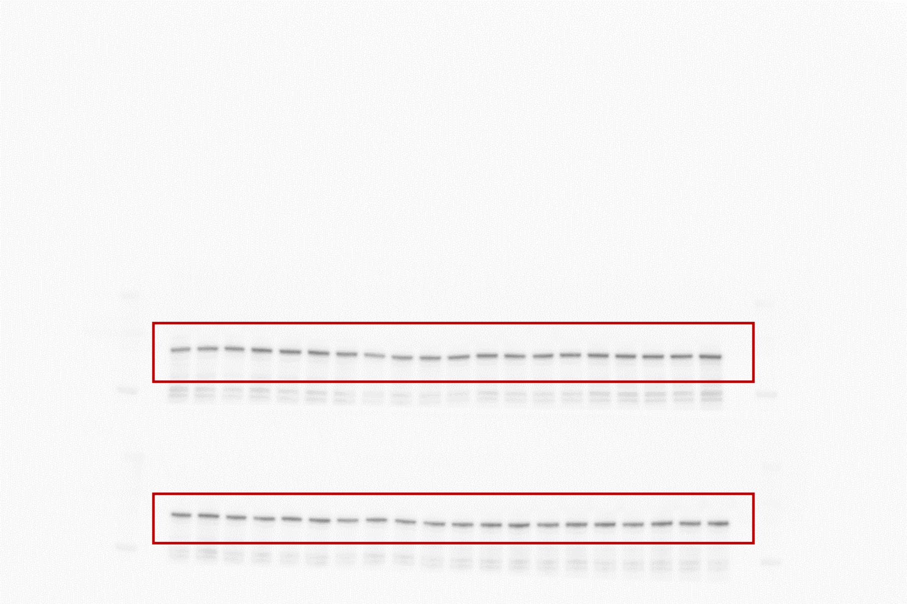

MPC-2

Hippocampus

membrane 1 and membrane 2

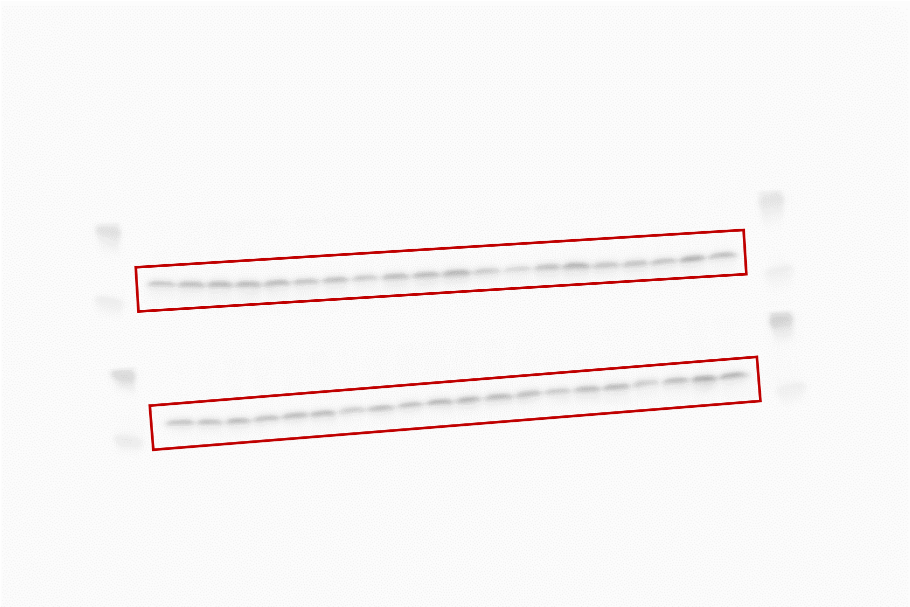

Samples from the left side - membrane 1 and 2:
CTRL, CTRL + STRESS, DEX, DEX + STRESS, CTRL, CTRL + STRESS, DEX, DEX + STRESS, CTRL, CTRL + STRESS, DEX, DEX + STRESS, CTRL, CTRL + STRESS, DEX, DEX + STRESS, CTRL, CTRL + STRESS, DEX, DEX + STRESS.

The results of the experiment are shown in Figure 8.

Vinculin as loading control to MPC-2

Hippocampus

membrane 1 and membrane 2

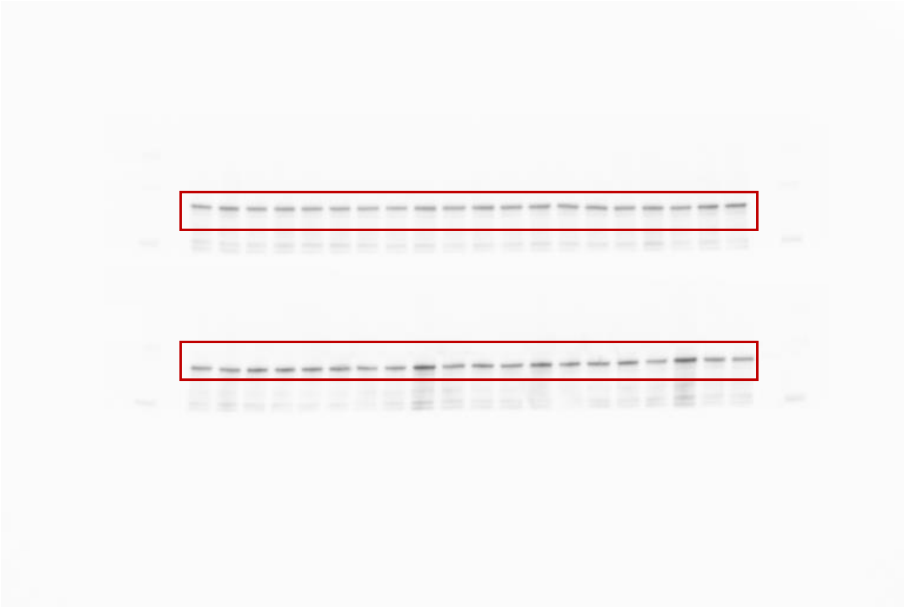

GPR81

Frontal cortex

membrane 1 and membrane 2

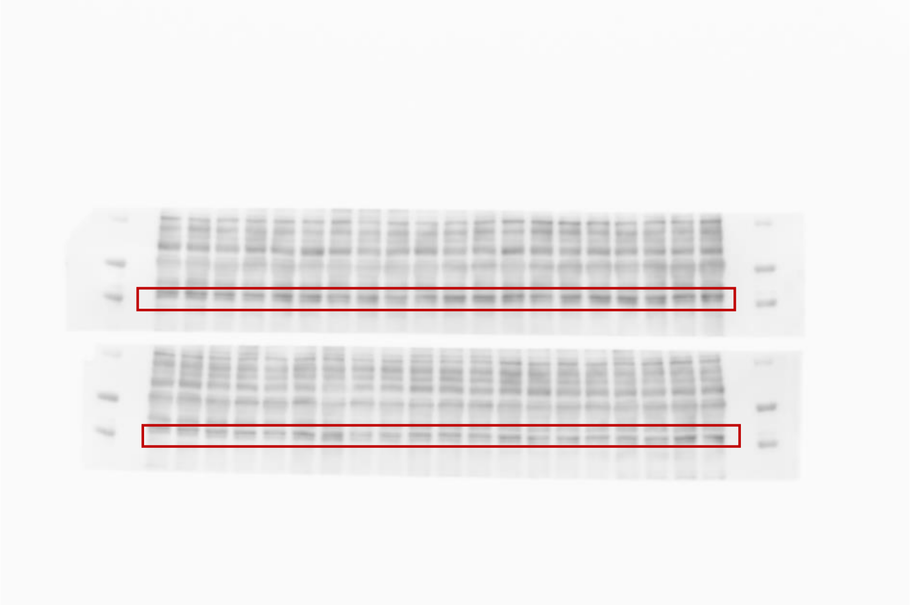

Samples from the left side - membrane 1 and 2:
CTRL, CTRL + STRESS, DEX, DEX + STRESS, CTRL, CTRL + STRESS, DEX, DEX + STRESS, CTRL, CTRL + STRESS, DEX, DEX + STRESS, CTRL, CTRL + STRESS, DEX, DEX + STRESS, CTRL, CTRL + STRESS, DEX, DEX + STRESS.

The results of the experiment are shown in Figure 9.

Vinculin as loading control to GPR81

Frontal cortex

membrane 1 and membrane 2

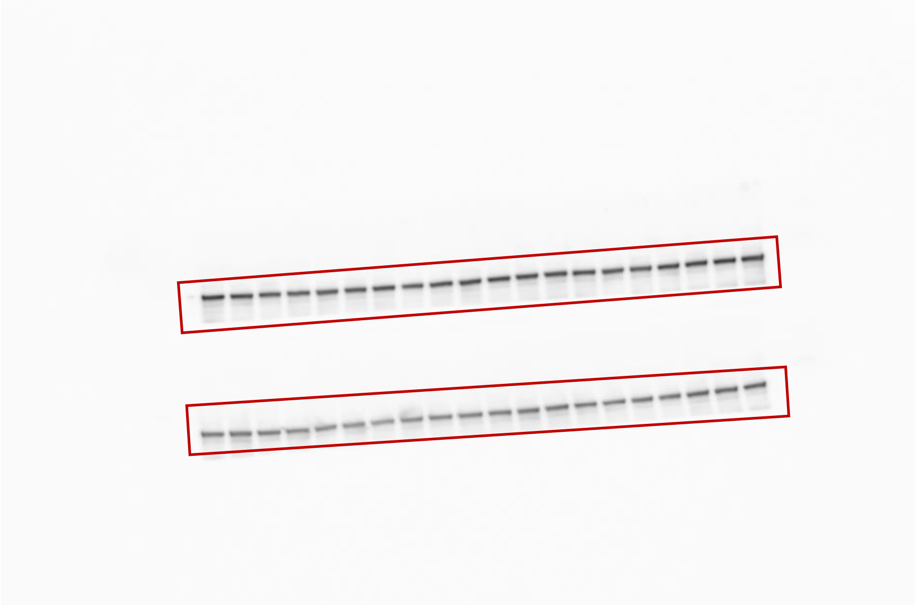

GPR81

Hippocampus

membrane 1 and membrane 2

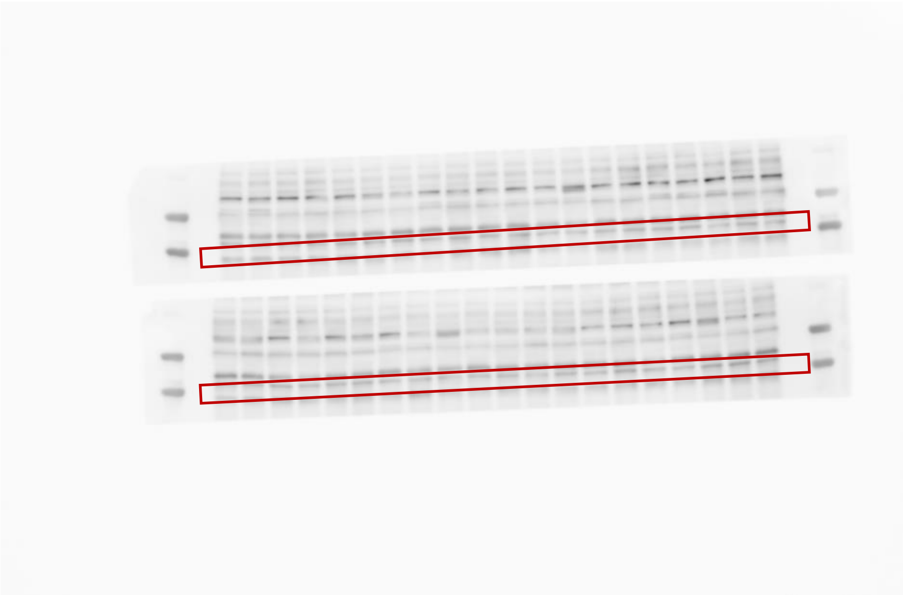

Samples from the left side - membrane 1 and 2:
CTRL, CTRL + STRESS, DEX, DEX + STRESS, CTRL, CTRL + STRESS, DEX, DEX + STRESS, CTRL, CTRL + STRESS, DEX, DEX + STRESS, CTRL, CTRL + STRESS, DEX, DEX + STRESS, CTRL, CTRL + STRESS, DEX, DEX + STRESS.

The results of the experiment are shown in Figure 9.

Vinculin as loading control to GPR81

Hippocampus

membrane 1 and membrane 2

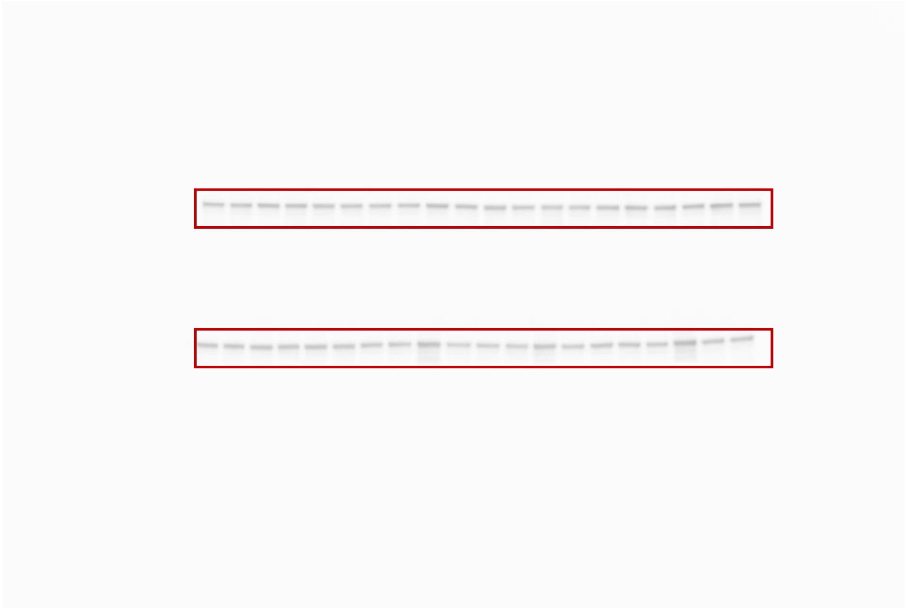

pAkt

Frontal cortex

membrane 1 and membrane 2

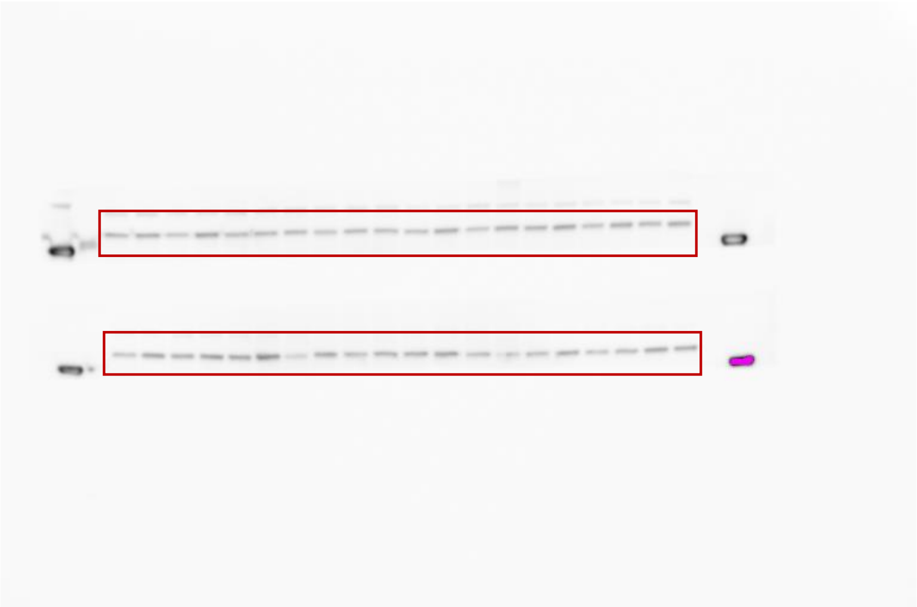

Akt

Frontal cortex

membrane 1 and membrane 2

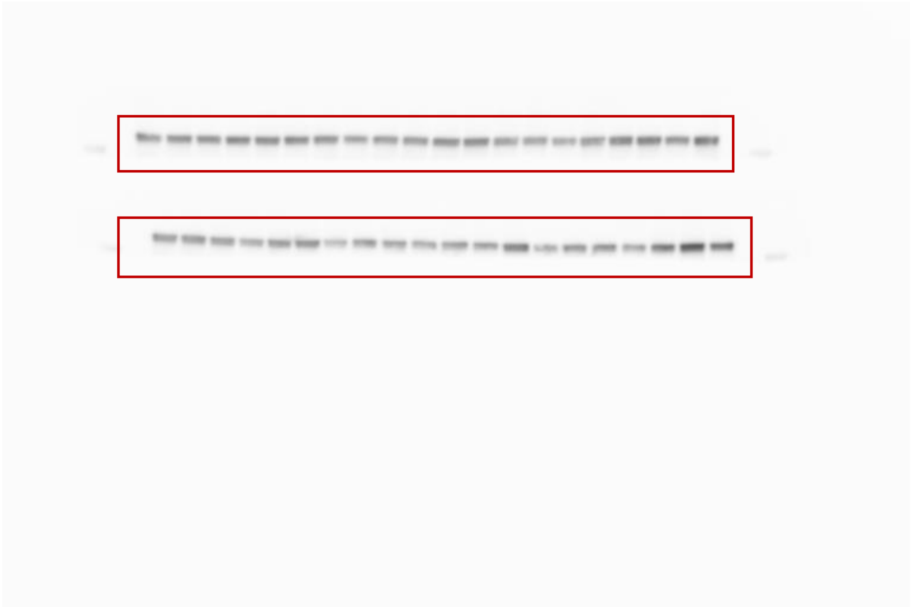

Samples from the left side - membrane 1 and 2:
CTRL, CTRL + STRESS, DEX, DEX + STRESS, CTRL, CTRL + STRESS, DEX, DEX + STRESS, CTRL, CTRL + STRESS, DEX, DEX + STRESS, CTRL, CTRL + STRESS, DEX, DEX + STRESS, CTRL, CTRL + STRESS, DEX, DEX + STRESS.

The results of the experiment are shown in Figure 9.

pAkt

Hippocampus

membrane 3 and membrane 4

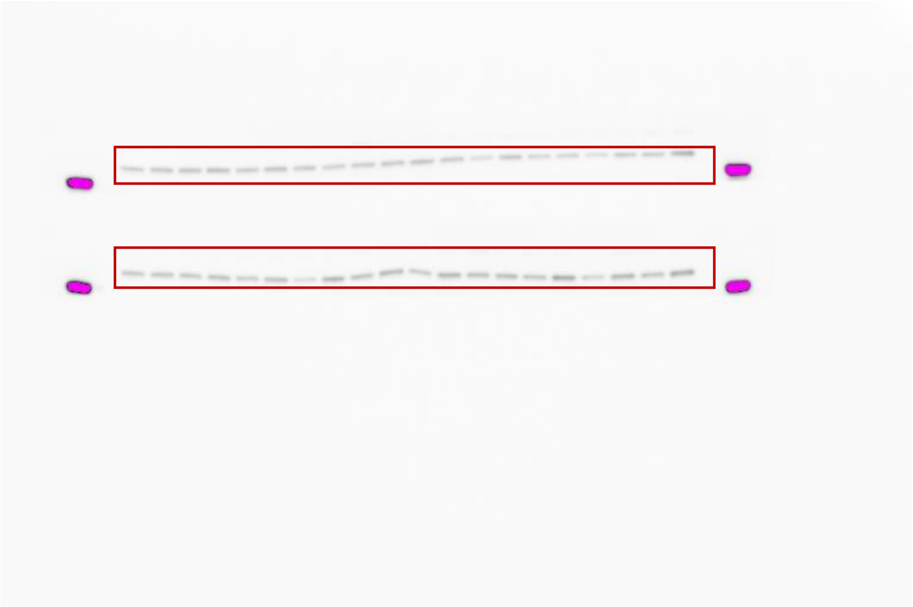

Samples from the left side - membrane 1 and 2:
CTRL, CTRL + STRESS, DEX, DEX + STRESS, CTRL, CTRL + STRESS, DEX, DEX + STRESS, CTRL, CTRL + STRESS, DEX, DEX + STRESS, CTRL, CTRL + STRESS, DEX, DEX + STRESS, CTRL, CTRL + STRESS, DEX, DEX + STRESS.

The results of the experiment are shown in Figure 11.

Akt

Hippocampus

membrane 3 and membrane 4

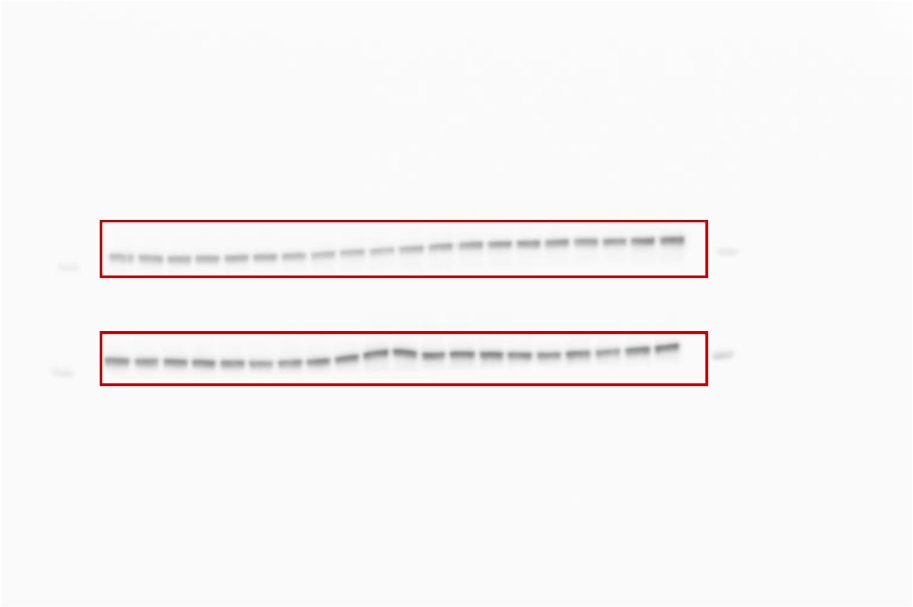

β-actin as loading control to Akt and pAkt

Frontal cortex

membrane 1 and membrane 2

Hippocampus

membrane 3 and membrane 4

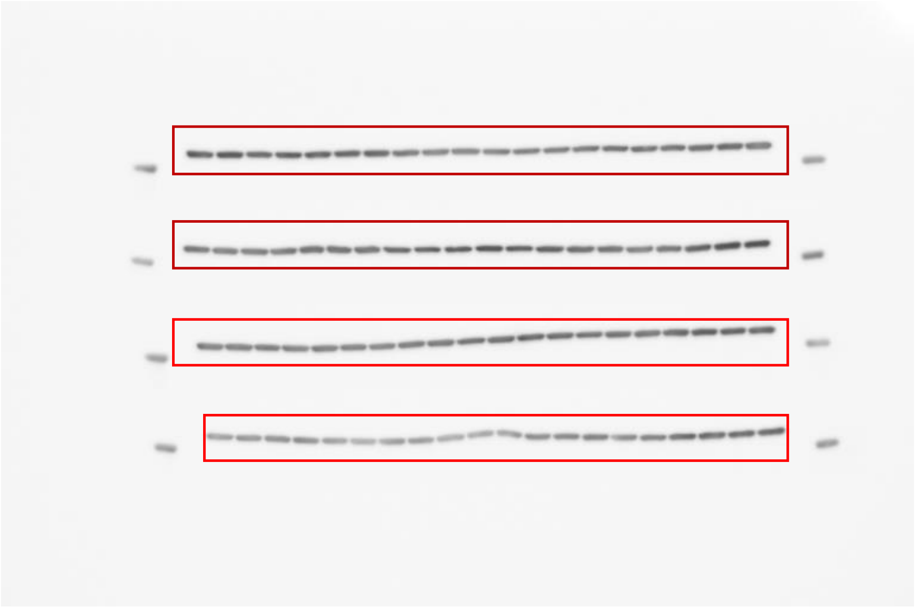

Samples from the left side - membrane 1-4:
CTRL, CTRL + STRESS, DEX, DEX + STRESS, CTRL, CTRL + STRESS, DEX, DEX + STRESS, CTRL, CTRL + STRESS, DEX, DEX + STRESS, CTRL, CTRL + STRESS, DEX, DEX + STRESS, CTRL, CTRL + STRESS, DEX, DEX + STRESS.

The results of the experiment are shown in Figure 11.

UCP-4

Frontal cortex

membrane 1 and membrane 2

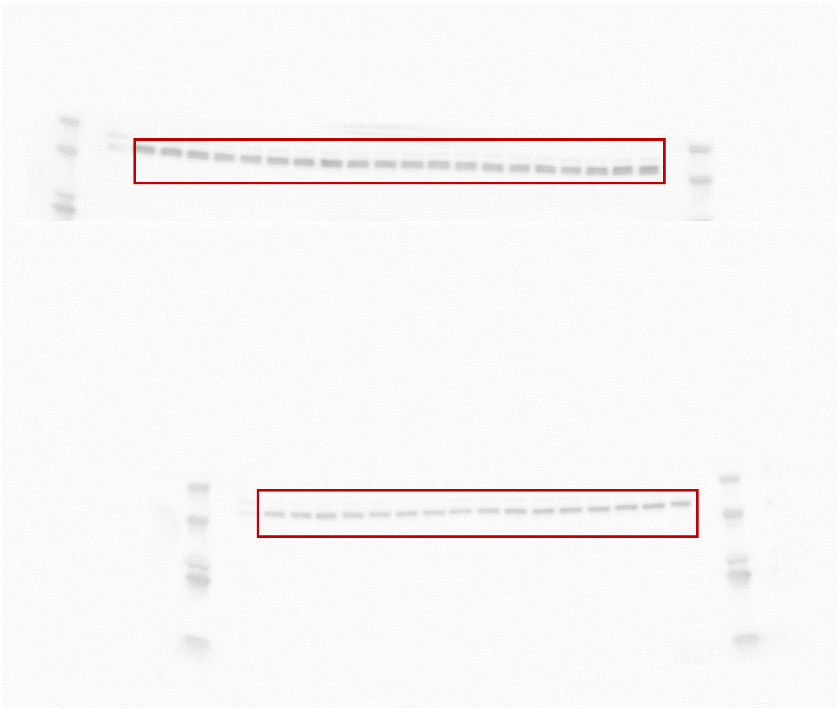

Samples from the left side - membrane 1:
CTRL, CTRL + STRESS, DEX, DEX + STRESS, CTRL, CTRL + STRESS, DEX, DEX + STRESS, CTRL, CTRL + STRESS, DEX, DEX + STRESS, CTRL, CTRL + STRESS, DEX, DEX + STRESS, CTRL, CTRL + STRESS, DEX, DEX + STRESS, CTRL, CTRL + STRESS, DEX, DEX + STRESS, CTRL, CTRL + STRESS, DEX, DEX + STRESS.

Samples from the left side - membrane 2:
CTRL, CTRL + STRESS, DEX, DEX + STRESS, CTRL, CTRL + STRESS, DEX, DEX + STRESS, CTRL, CTRL + STRESS, DEX, DEX + STRESS, CTRL, CTRL + STRESS, DEX, DEX + STRESS.

The results of the experiment are shown in Table 1.

β-actin as loading control to UCP-4

Frontal cortex

membrane 1 and membrane 2

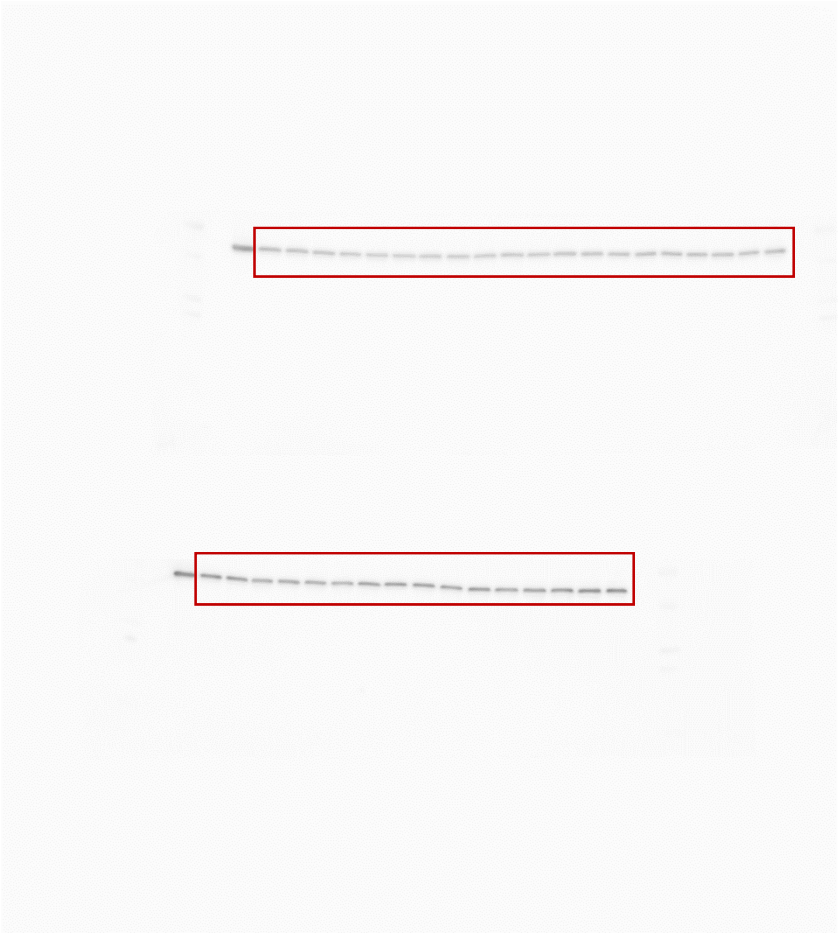

UCP-4

Hippocampus

membrane 1 and membrane 2

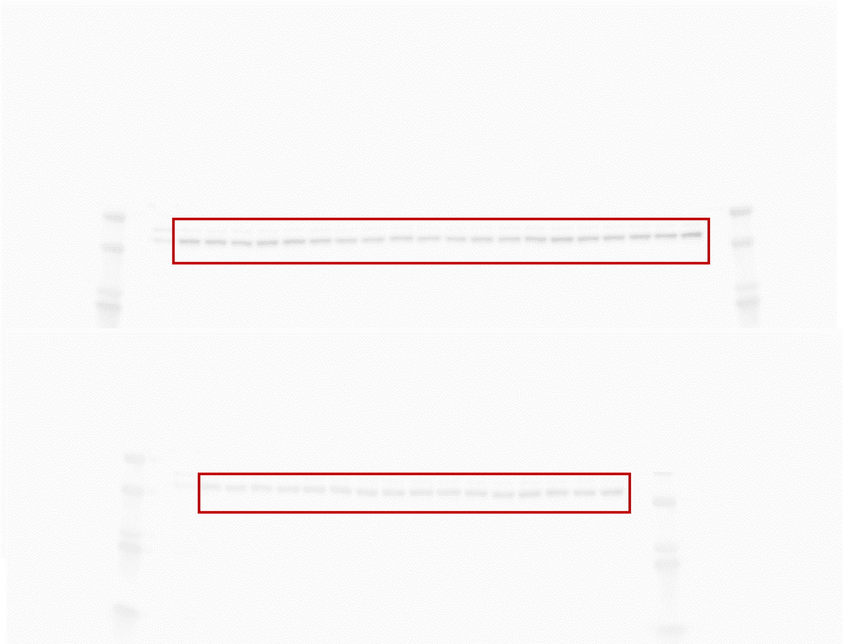

Samples from the left side - membrane 1:
CTRL, CTRL + STRESS, DEX, DEX + STRESS, CTRL, CTRL + STRESS, DEX, DEX + STRESS, CTRL, CTRL + STRESS, DEX, DEX + STRESS, CTRL, CTRL + STRESS, DEX, DEX + STRESS, CTRL, CTRL + STRESS, DEX, DEX + STRESS.

Samples from the left side - membrane 2:
CTRL, CTRL + STRESS, DEX, DEX + STRESS, CTRL, CTRL + STRESS, DEX, DEX + STRESS, CTRL, CTRL + STRESS, DEX, DEX + STRESS, CTRL, CTRL + STRESS, DEX, DEX + STRESS.

The results of the experiment are shown in Table 1.

β-actin as loading control to UCP-4

Hippocampus

membrane 1 and membrane 2

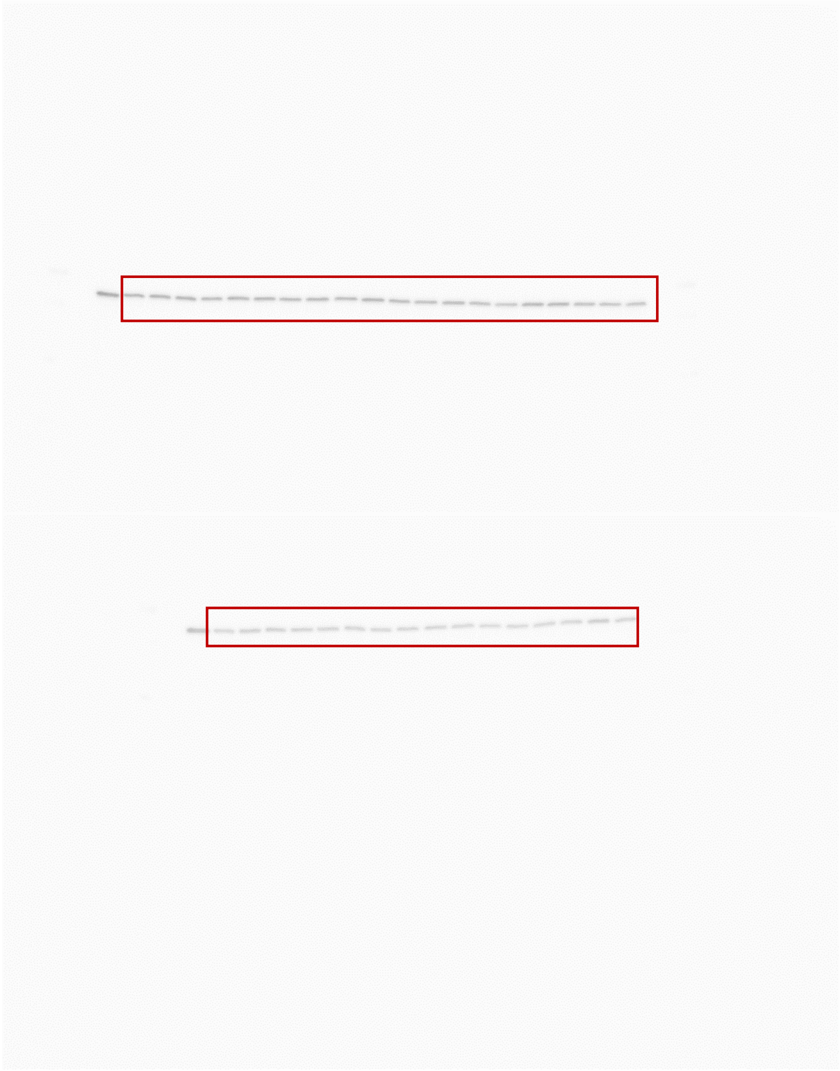

MFN2

Frontal cortex

membrane 1 and membrane 2

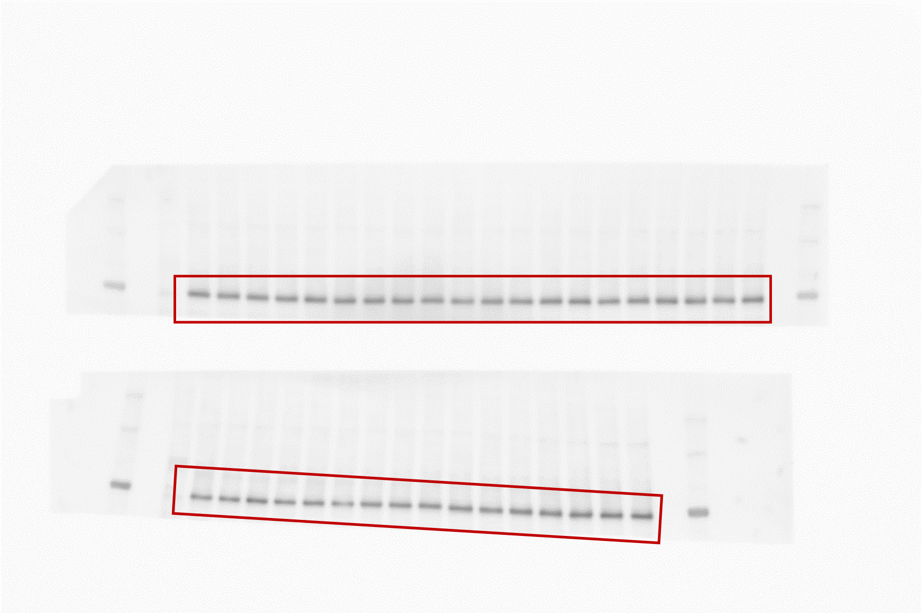

Samples from the left side - membrane 1:
CTRL, CTRL + STRESS, DEX, DEX + STRESS, CTRL, CTRL + STRESS, DEX, DEX + STRESS, CTRL, CTRL + STRESS, DEX, DEX + STRESS, CTRL, CTRL + STRESS, DEX, DEX + STRESS, CTRL, CTRL + STRESS, DEX, DEX + STRESS.

Samples from the left side - membrane 2:
CTRL, CTRL + STRESS, DEX, DEX + STRESS, CTRL, CTRL + STRESS, DEX, DEX + STRESS, CTRL, CTRL + STRESS, DEX, DEX + STRESS, CTRL, CTRL + STRESS, DEX, DEX + STRESS.

The results of the experiment are shown in Table 1.

β-actin as loading control to MFN2

Frontal cortex

membrane 1 and membrane 2

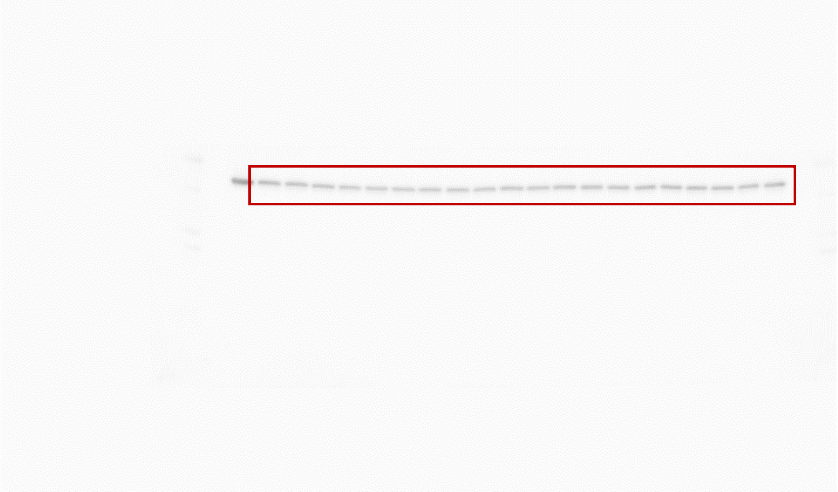

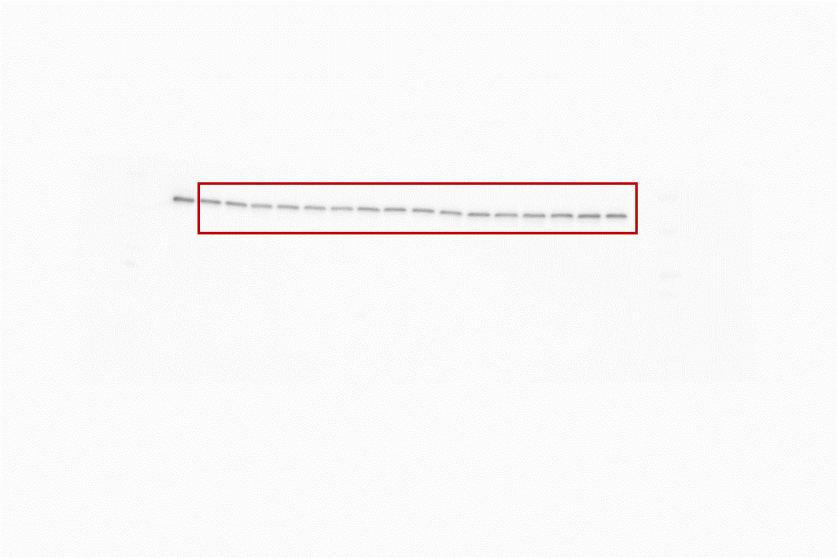

MFN2

Hippocampus

membrane 1 and membrane 2

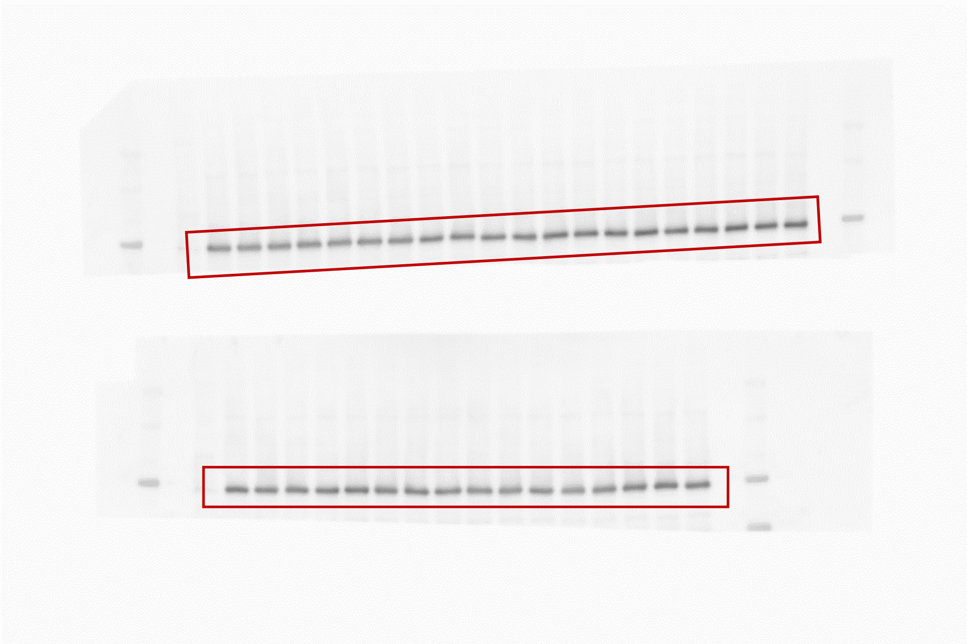

Samples from the left side - membrane 1:
CTRL, CTRL + STRESS, DEX, DEX + STRESS, CTRL, CTRL + STRESS, DEX, DEX + STRESS, CTRL, CTRL + STRESS, DEX, DEX + STRESS, CTRL, CTRL + STRESS, DEX, DEX + STRESS, CTRL, CTRL + STRESS, DEX, DEX + STRESS.

Samples from the left side - membrane 2:
CTRL, CTRL + STRESS, DEX, DEX + STRESS, CTRL, CTRL + STRESS, DEX, DEX + STRESS, CTRL, CTRL + STRESS, DEX, DEX + STRESS, CTRL, CTRL + STRESS, DEX, DEX + STRESS.

The results of the experiment are shown in Table 1.

β-actin as loading control to MFN2

Hippocampus

membrane 1 and membrane 2

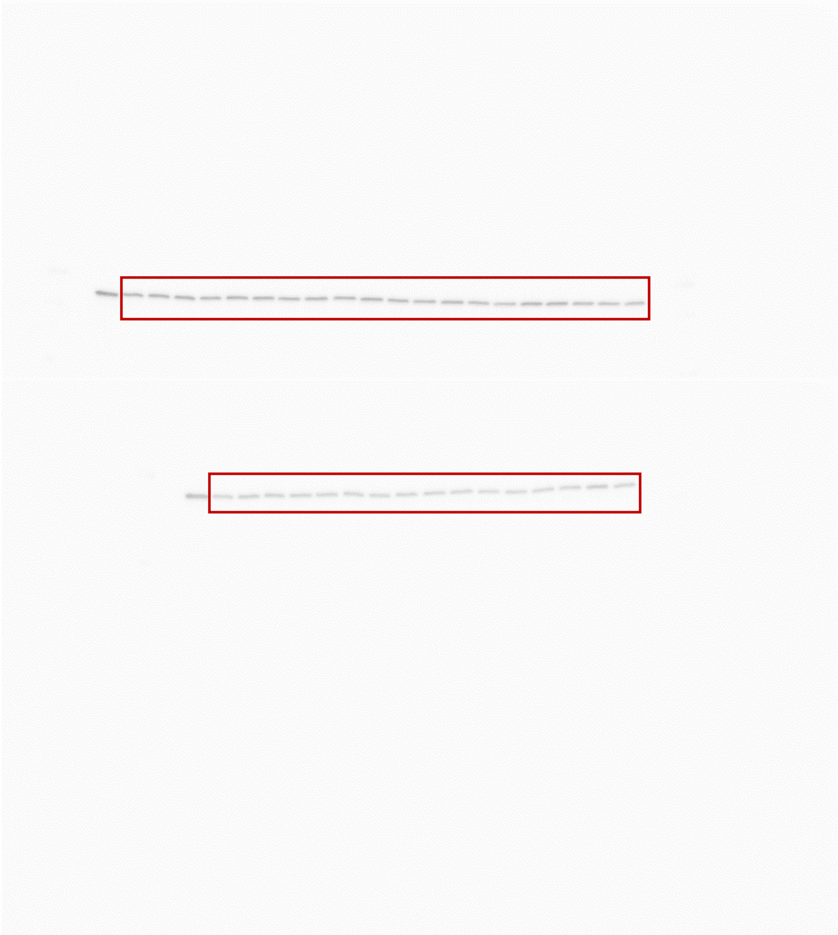

OPA1

Frontal cortex

membrane 1 and membrane 2

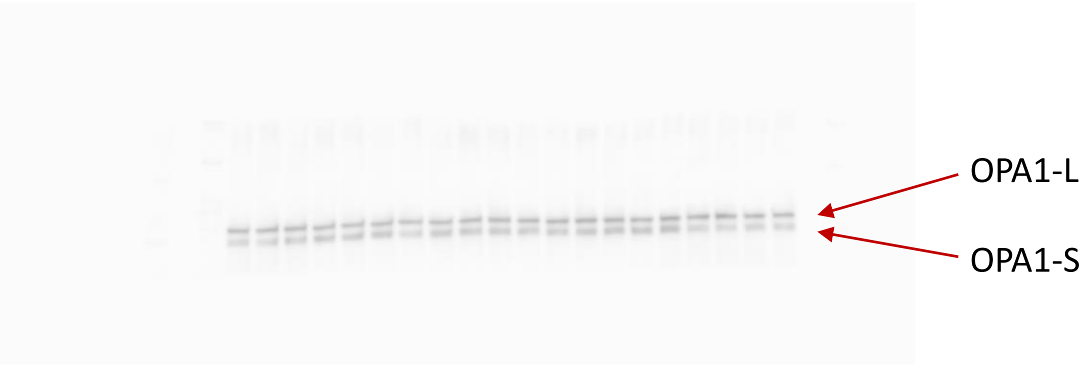

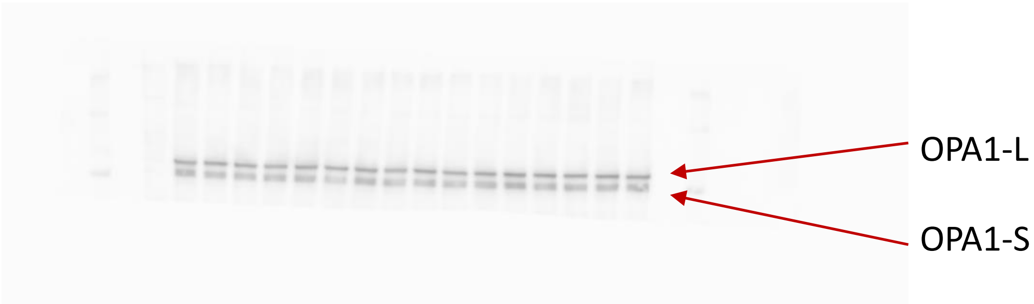

Samples from the left side - membrane 1:
CTRL, CTRL + STRESS, DEX, DEX + STRESS, CTRL, CTRL + STRESS, DEX, DEX + STRESS, CTRL, CTRL + STRESS, DEX, DEX + STRESS, CTRL, CTRL + STRESS, DEX, DEX + STRESS, CTRL, CTRL + STRESS, DEX, DEX + STRESS.

Samples from the left side - membrane 2:
CTRL, CTRL + STRESS, DEX, DEX + STRESS, CTRL, CTRL + STRESS, DEX, DEX + STRESS, CTRL, CTRL + STRESS, DEX, DEX + STRESS, CTRL, CTRL + STRESS, DEX, DEX + STRESS.

The results of the experiment are shown in Table 1.

β-actin as loading control to OPA1

Frontal cortex

membrane 1 and membrane 2

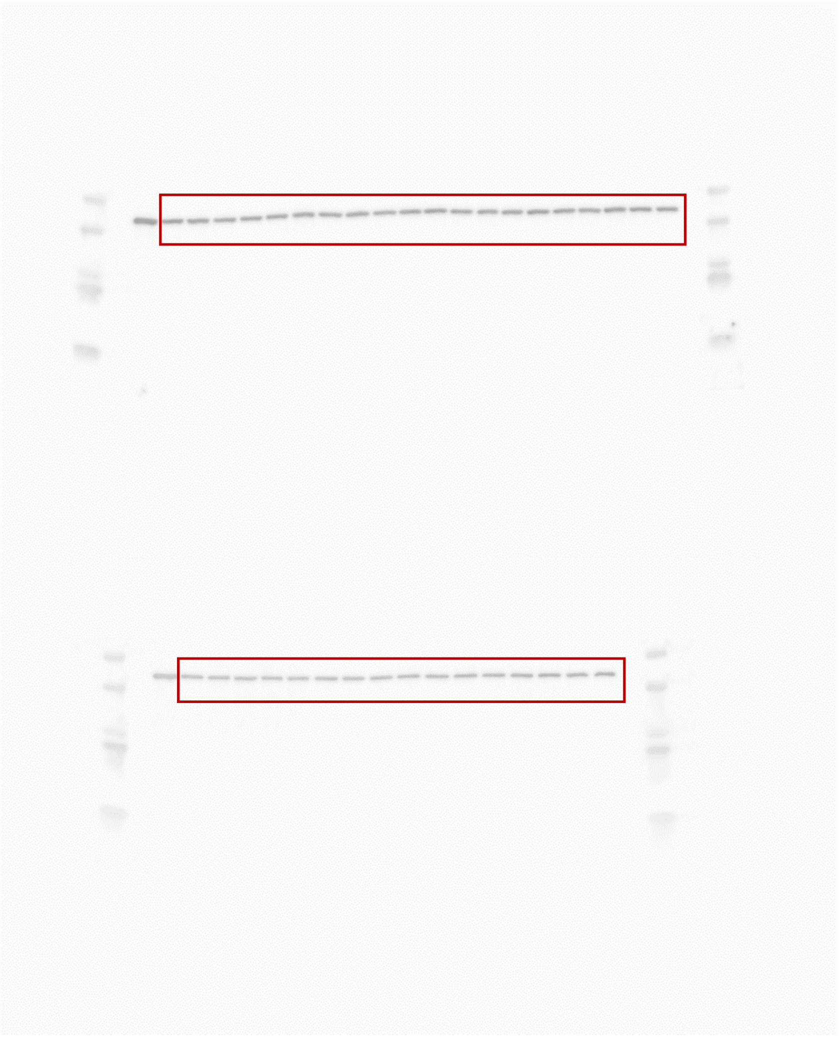

OPA1

Hippocampus

membrane 1 and membrane 2

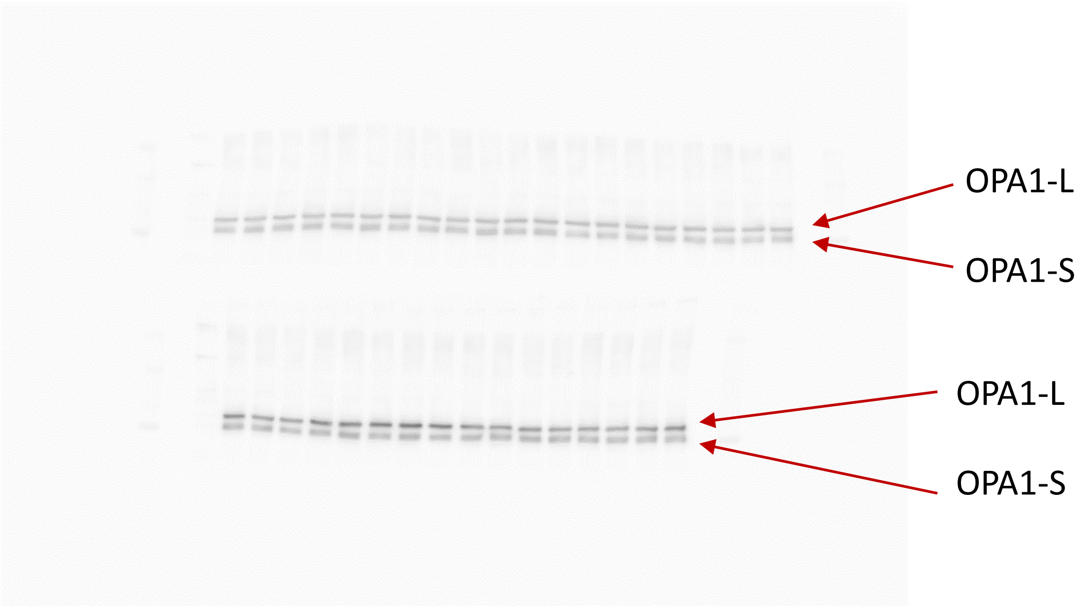

Samples from the left side - membrane 1:
CTRL, CTRL + STRESS, DEX, DEX + STRESS, CTRL, CTRL + STRESS, DEX, DEX + STRESS, CTRL, CTRL + STRESS, DEX, DEX + STRESS, CTRL, CTRL + STRESS, DEX, DEX + STRESS, CTRL, CTRL + STRESS, DEX, DEX + STRESS.

Samples from the left side - membrane 2:
CTRL, CTRL + STRESS, DEX, DEX + STRESS, CTRL, CTRL + STRESS, DEX, DEX + STRESS, CTRL, CTRL + STRESS, DEX, DEX + STRESS, CTRL, CTRL + STRESS, DEX, DEX + STRESS.

The results of the experiment are shown in Table 1.

β-actin as loading control to OPA1

Hippocampus

membrane 1 and membrane 2

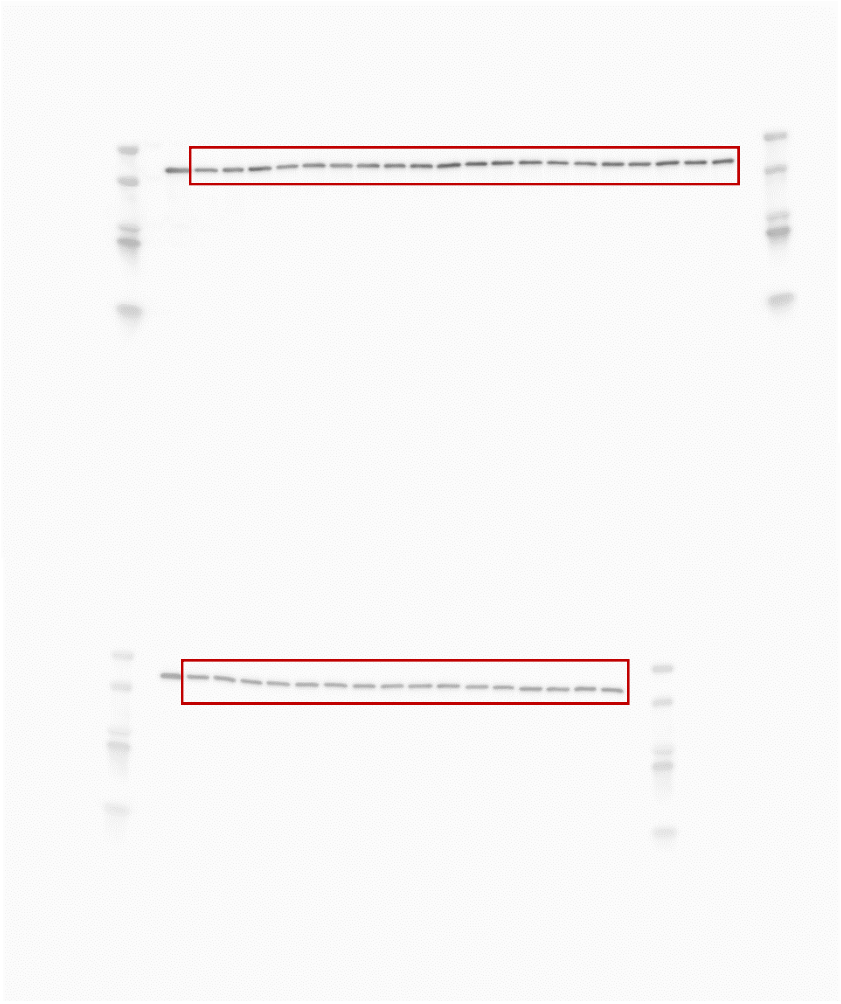

Not all WB samples (presented on pages 2-24) were analyzed (poor quality or outliers were not analyzed).
